# Machine learning driven web-based app platform for the discovery of monoamine oxidase B inhibitors

**DOI:** 10.1038/s41598-024-55628-y

**Published:** 2024-02-28

**Authors:** Sunil Kumar, Ratul Bhowmik, Jong Min Oh, Mohamed A. Abdelgawad, Mohammed M. Ghoneim, Rasha Hamed Al‑Serwi, Hoon Kim, Bijo Mathew

**Affiliations:** 1 Department of Pharmaceutical Chemistry, Amrita School of Pharmacy, Amrita Vishwa Vidyapeetham, AIMS Health Sciences Campus, Kochi, India; 2 Department of Pharmaceutical Chemistry, School of Pharmaceutical Education and Research, Jamia Hamdard, New Delhi, India; 3 Department of Pharmacy, and Research Institute of Life Pharmaceutical Sciences, Sunchon National University, Suncheon, 57922 Republic of Korea; 4 Department of Pharmaceutical Chemistry, College of Pharmacy, Jouf University, 72341 Sakaka, Aljouf Saudi Arabia; 5 Department of Pharmacy Practice, College of Pharmacy, AlMaarefa University, 13713 Ad Diriyah, Riyadh Saudi Arabia; 6 Department of Basic Dental Sciences, College of Dentistry, Princess Nourah Bint Abdulrahman University, P.O. Box 84428, 11671 Riyadh, Saudi Arabia

**Keywords:** Monoamine oxidase B, ML-QSAR, PubChem fingerprints, Substructure fingerprints, 1D and 2D molecular descriptors, Prediction models, Bioactivity, Web application, Molecular docking, Molecular interactions, Molecular dynamics simulation, Biochemistry, Biological techniques, Drug discovery

## Abstract

Monoamine oxidases (MAOs), specifically MAO-A and MAO-B, play important roles in the breakdown of monoamine neurotransmitters. Therefore, MAO inhibitors are crucial for treating various neurodegenerative disorders, including Parkinson's disease (PD), Alzheimer’s disease (AD), and amyotrophic lateral sclerosis (ALS). In this study, we developed a novel cheminformatics pipeline by generating three diverse molecular feature-based machine learning-assisted quantitative structural activity relationship (ML-QSAR) models concerning MAO-B inhibition. PubChem fingerprints, substructure fingerprints, and one-dimensional (1D) and two-dimensional (2D) molecular descriptors were implemented to unravel the structural insights responsible for decoding the origin of MAO-B inhibition in 249 non-reductant molecules. Based on a random forest ML algorithm, the final PubChem fingerprint, substructure fingerprint, and 1D and 2D molecular descriptor prediction models demonstrated significant robustness, with correlation coefficients of 0.9863, 0.9796, and 0.9852, respectively. The significant features of each predictive model responsible for MAO-B inhibition were extracted using a comprehensive variance importance plot (VIP) and correlation matrix analysis. The final predictive models were further developed as a web application, MAO-B-pred (https://mao-b-pred.streamlit.app/), to allow users to predict the bioactivity of molecules against MAO-B. Molecular docking and dynamics studies were conducted to gain insight into the atomic-level molecular interactions between the ligand-receptor complexes. These findings were compared with the structural features obtained from the ML-QSAR models, which supported the mechanistic understanding of the binding phenomena. The presented models have the potential to serve as tools for identifying crucial molecular characteristics for the rational design of MAO-B target inhibitors, which may be used to develop effective drugs for neurodegenerative disorders.

## Introduction

Monoamine oxidase (MAO)-A and MAO-B are the enzymes located in the mitochondria. They break down monoamine neurotransmitters, such as adrenaline, noradrenaline, serotonin, norepinephrine, β-phenylethylamine, dopamine (DA), and dietary amines like tyramine^1,2^. Both isozymes have distinct substrate and inhibitor specificities; MAO-A demonstrates greater affinity for hydroxylated amines, such as noradrenaline and serotonin, whereas MAO-B interacts with non-hydroxylated amines, such as benzylamine and beta-phenylethylamine. Nevertheless, both DA and tyramine exhibited comparable affinities for isoform^3^. Overexpression of MAO enzymes can lead to mitochondrial damage, depression, Alzheimer's disease (AD), Parkinson's disease (PD), and other conditions owing to the neurotoxic nature of their metabolites, including hydrogen peroxide, ammonia, and different aldehydes. The depletion of DA, noradrenaline, and serotonin serves as the molecular basis for the underlying degenerative processes in PD. Consistent with this concept, the discovery of small molecules that can block MAOs has led to considerable advancements in the treatment of various neuropsychiatric disorders and neurodegenerative diseases such as PD and AD. Numerous MAO inhibitors have been developed since the 1960s, with phenelzine, tranylcypromine, pargyline, selegiline, clorgyline, moclobemide, rasagiline, safinamide, and ladostigil being the most therapeutically important medications^1–6^. The positive effect of selegiline on patients with PD has led to the initiation of extensive pharmacological research initiatives for neuropsychiatric diseases, focusing on MAO inhibitors. Advancements in selective irreversible inhibitors of MAOs have helped partially clarify the challenges associated with the clinical use of MAO inhibitors. The administration of MAO inhibitors is associated with several factors, such as health improvement, mental and neurological diseases, off-target effects, safety concerns, dietary restrictions, and, specifically, low tyramine intake. The pharmacological effects of tyramine are derived from its specific inhibition of MAO-A over MAO-B^1,3,7^.

The specificity of the small-molecule inhibitors targeting MAO-A and MAO-B was determined by analyzing their three-dimensional (3D) structures when co-crystallized with an inhibitor. Although a two-site cavity structure consisting of an entering site and a reactive site cavity was observed for MAO-B, the active site of MAO-A was distinct, shorter, and wider than the elongated and narrower substrate pockets in MAO-B. Knowledge acquired from the three-dimensional structures of the binding sites of MAOs has been used to create new reversible and irreversible inhibitors. Recently, these inhibitors have been used to treat both affective and neurological illnesses. Several reversible inhibitors of MAO-A (RIMA) that do not cause cheese effects have been developed; however, only moclobemide has been authorized for the treatment of depression. MAO-B inhibitors, which have received clinical approval for the treatment of PDs, are classified into numerous chemical classes, including hydrazine, cyclopropylamine, and propargylamine. Three drugs, selegiline and rasagiline, belong to the propargylamine class and exhibit some selectivity for MAO-B. Safinamide is a selective and reversible inhibitor that is specifically used for the treatment of PD. Nevertheless, most MAO inhibitors exhibit limited specificity, necessitating the continuous development of more selective inhibitors using accumulated knowledge to develop logical design techniques^1,3^. Enhancing the ability to promptly detect off-target interactions is crucial for reducing the rate of drug attrition owing to safety concerns during clinical testing, as the absence of selectivity can lead to harmful toxicities. The therapeutic potential of MAO inhibitors, along with a comprehensive understanding of the 3D structure of the MAO-B active site, could greatly enhance progress in creating new medications for PD. Despite the ongoing synthesis and clinical testing of various compounds as potential treatments for PD, the emergence of dietary restrictions and adverse effects has necessitated the development of new theoretical, experimental, and therapeutic approaches to address these limitations.

**Figure 1:**
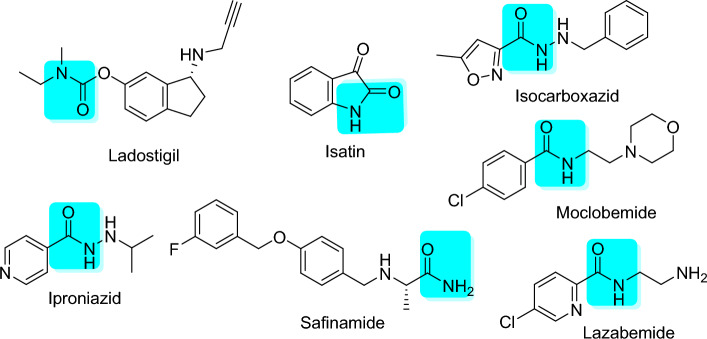
Structures of amide-based MAO inhibitors.

Hagenow et al. demonstrated that ligands with two aryl moieties joined by short spacers are strong inhibitors of MAO-B and are good targets for the treatment of neurological disorders^8^. Numerous spacers, such as amides, benzyloxies, conjugated dienones, chalcones, hydrazones, pyrazolines, and styryl-based frameworks, are considered effective for designing MAO-B inhibitors^4,9–12^. Amides have a great deal of structural variability that enables medicinal chemists to create compounds with qualities essential for the development of central nervous system (CNS) drugs. Compounds with the desired pharmacological characteristics can be designed with flexibility, owing to the ability of the amide functional group to be integrated into a variety of molecular frameworks. Hydrogen bonds can be formed by amides, which are essential for interactions with biological molecules, such as receptors and enzymes. In summary, amides play a crucial role in the discovery and design of CNS-active drugs owing to their unique ability to interact with certain biological targets, optimize pharmacokinetics, and fine-tune their structures. A wide range of MAO inhibitors such as isocarboxazid, iproniazid, moclobemide, lazabemide, ladostigil, safinamide, and isatin (Fig. 1) contain amide linkers. We investigated amides using the QSAR model to determine the significance of their chemical characteristics in MAO inhibition.

**Figure 2:**
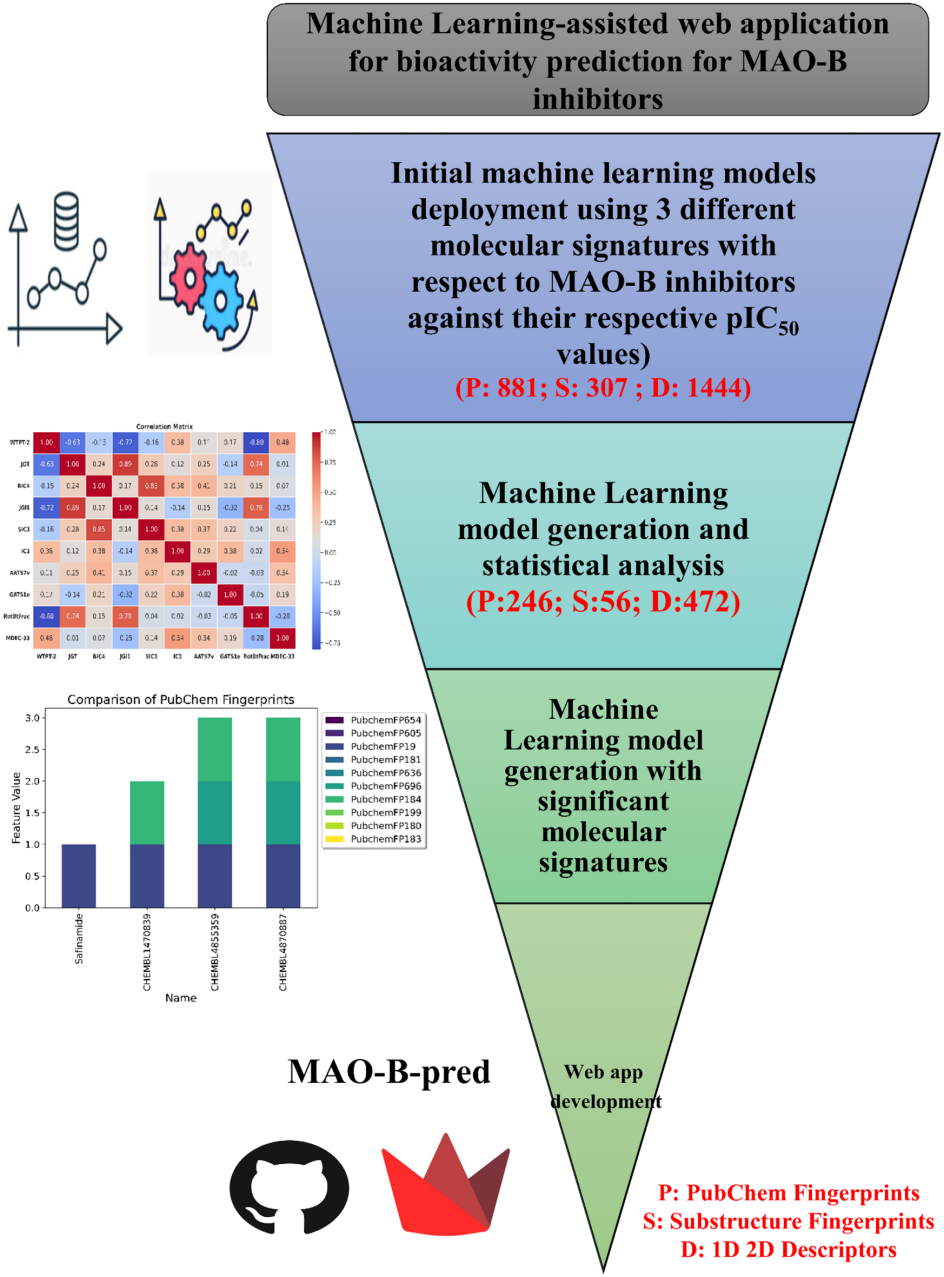
Workflow for the generation of multi-feature ML-QSAR predictive models.

Computational methods, including quantitative structure–activity relationships (QSAR), pharmacophore modeling, molecular docking, and molecular dynamics (MD) simulations, are crucial for the development and identification of novel drugs with improved therapeutic effects^13–21^. Cheminformatics and molecular modeling methods have been used for numerous years to discover and develop new drugs with improved therapeutic properties in several areas. Currently*, *in silico modeling is an integral component of standard drug discovery processes. These methods are typically used to identify new medications or enhance the therapeutic effectiveness of a chemical series during the early stages of drug development^18,20,22^. Our study proposes a novel cheminformatics pipeline to generate multiple machine learning-assisted quantitative structural activity relationship (ML-QSAR) prediction models with diverse molecular features to explore the chemical space of MAO-B inhibitors. Robust ML-QSAR models were further implemented as a Python web application, MAO-B-pred (https://mao-b-pred.streamlit.app/), which provides a user-friendly interface that can predict the inhibitory activity of small molecules against MAO-B^23–26^. This web application has the advantage of being platform independent and can be accessed through a web browser on any operating system or device. Subsequently, we implemented this web application to screen large oxidoreductase chemical libraries to identify potent MAO-B inhibitors. In addition, we conducted molecular docking and simulation analyses using the screened oxidoreductase molecules with a predicted pIC_50_ ≥ 6 (IC_50_: 100–1000 nM) to elucidate the effects of various features observed in the ML-QSAR models. This study identified several key structural attributes of small molecule compounds that can inhibit MAO-B receptors. Additionally, we investigated the correlation between these features and the interactions between molecules and their target receptors. Acquiring this understanding will facilitate the development of advanced treatments for PD with respect to MAO-B inhibition mechanisms (Fig. 2).

## Results and discussion

### Selection of ligands

A total of 249 amide-based MAO-B inhibitors were retrieved from the existing literature up to 2023, along with their structures in the Simplified Molecular Input Line Entry System (SMILES) notation and IC_50_ values in nM. To achieve a more uniform distribution of the IC_50_ data, the IC_50_ values of the 249 molecules were converted to a negative logarithmic scale, referred to as pIC_50_, and represented as -log_10_(IC_50_).

### Exploratory data analysis

**Figure 3:**
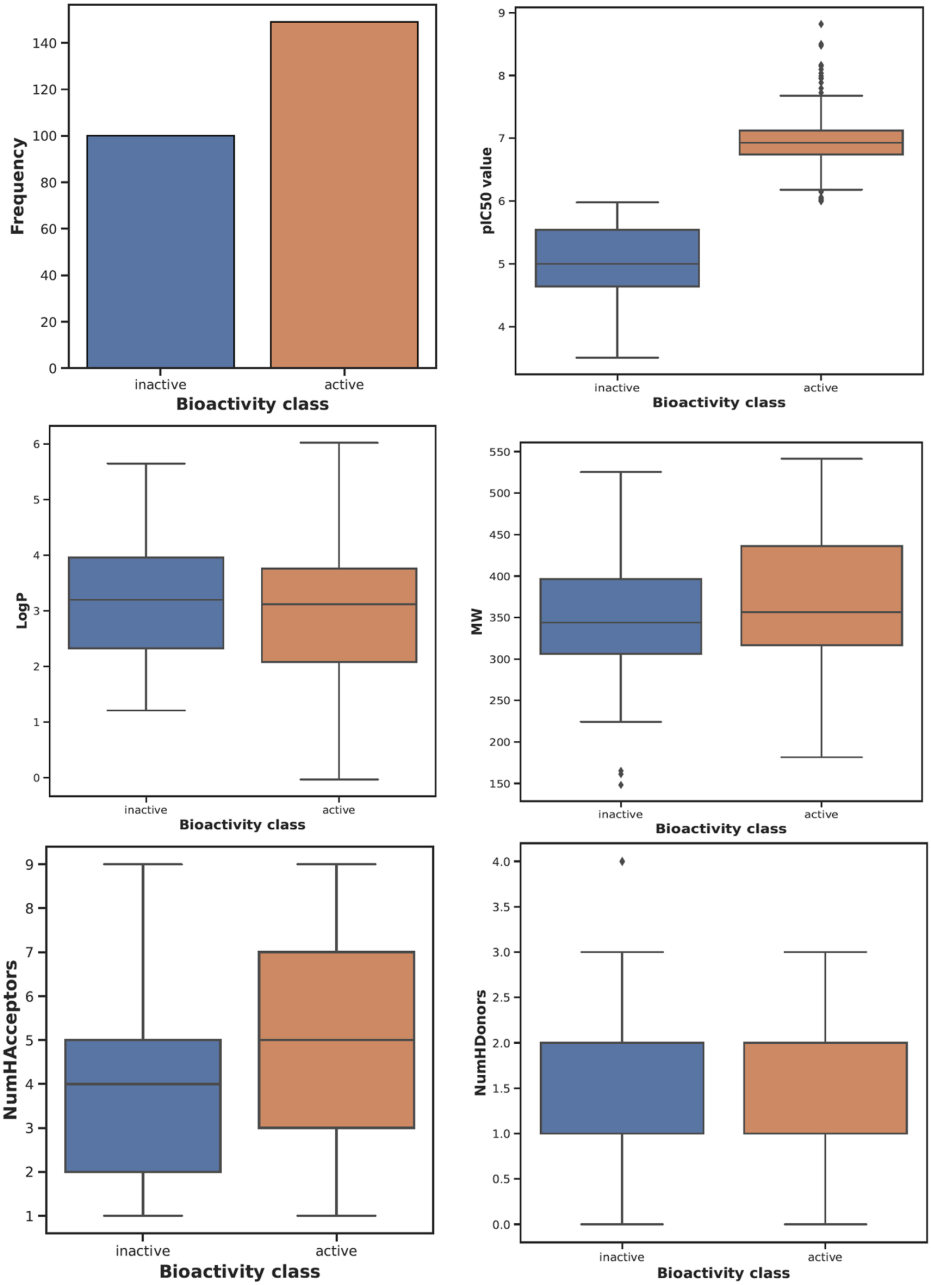
Exploratory data analysis of the curated amide-based MAO-B inhibitor dataset.

The RDkit software was further utilized to compute Lipinski’s rule of five molecular descriptors, including molecular weight, Log P, hydrogen bond acceptor count, and hydrogen bond donor count for all 249 molecules. The Matplotbib and seaborn packages were used to graphically explore the correlation of Lipinski’s descriptors with bioactivity (pIC_50_) values. Graphical bar plots demonstrate that the numerical count of active molecules against MAO-B was significantly greater in our dataset than that of the inactive compounds. Furthermore, the range of pIC_50_ values for the active molecules in our dataset was determined to range 6.0–8.8, whereas for the inactive molecules, the pIC_50_ value was determined to be < 5. The log *P* values for the active and inactive molecules were determined to be in the ranges 0.1–6.0 and 1.2–5.6, respectively, whereas the ranges of molecular weight (MW) for the active and inactive molecules were observed to be in the ranges 180–540 and 230–530 Da (Da), respectively. The presence of hydrogen bond acceptors and donors was shown to be similar in both the active and inactive molecules, that is, within the range of nine and three, respectively. Chemical space analysis revealed that the active molecules in our curated dataset followed Lipinski’s rule of five (Fig. 3).

### Molecular feature exploration

We implemented the PaDELPy Python wrapper (https://github.com/ecrl/padelpy) in PaDEL software to calculate three different molecular features for our 249 MAO-B inhibitors. We individually calculated PubChem fingerprints, substructure fingerprints, and 1D and 2D molecular descriptors for each of the 249 molecules and created individual datasets for each feature, along with their respective pIC_50_ values and their respective SMILES notations. For the PubChem fingerprint feature collection, we initially calculated 881 molecular features. Using the variance threshold method, 246 PubChem fingerprints were shown to be significant. For the substructure fingerprint feature collection, 307 features were initially calculated. Furthermore, we used the variance threshold method to extract 56 significant substructure fingerprints from the 307 initial fingerprints. Regarding the 1D and 2D molecular descriptor feature collection, we initially computed a total of 1,444 molecular characteristics. Using a variance threshold approach, 472 significant molecular descriptors were identified.

### ML-QSAR model optimization using applicability domain analysis

**Figure 4:**
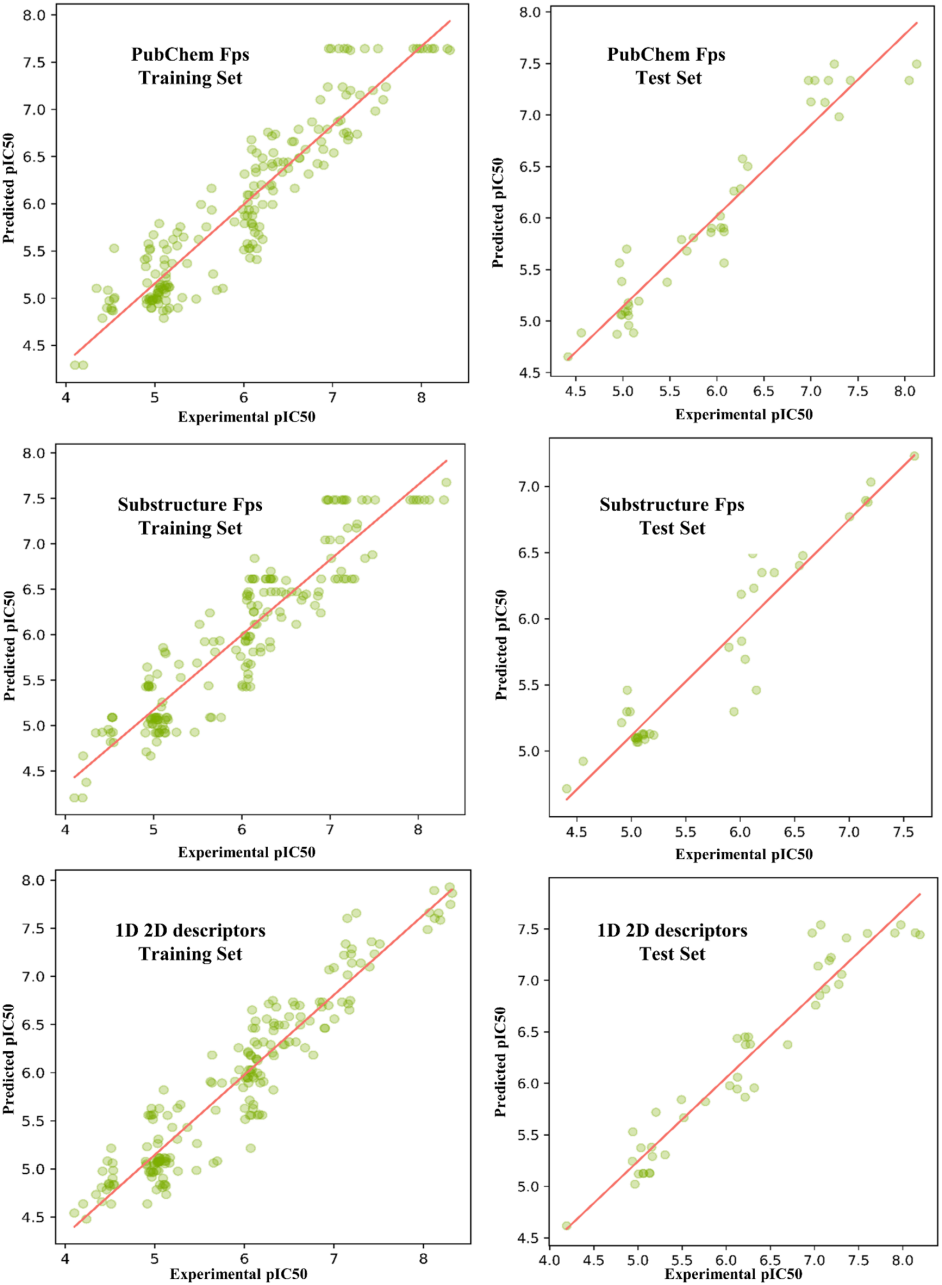
Regression plots of all generated ML-QSAR models.

**Figure 5:**
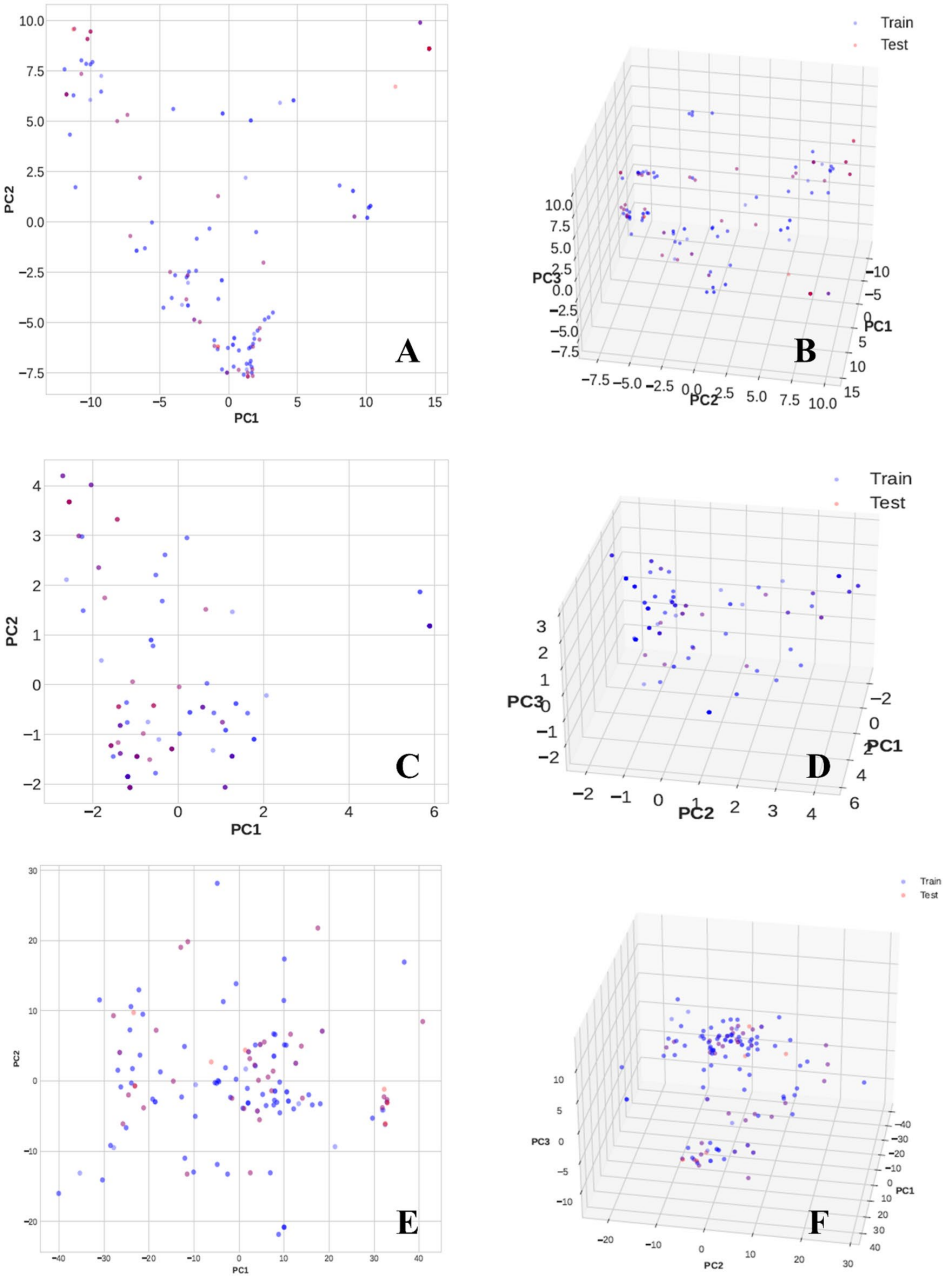
Applicability domain analysis by PCA plot for the generated ML-QSAR models. (**A**) and (**B**) show 2D and 3D PCA of the PubChem fingerprint prediction model; (**C**) and (**D**) show 2D and 3D PCA of the substructure fingerprint prediction model; (**E**) and (**F**) show 2D and 3D analyses of the 1D and 2D molecular descriptor prediction model.

We conducted applicability domain analysis to identify and remove outliers from each ML-QSAR prediction model. Using a random forest-based PubChem fingerprint prediction model, we identified and removed 25 molecules using principal component analysis (PCA). Our optimized random-forest-based PubChem fingerprint model demonstrated correlation coefficients of 0.9863 and 0.9397, RMSEs of 0.212 and 0.2971, and mean absolute errors (MAEs) of 0.1645 and 0.2389 for the training and test sets of 168 and 56 molecules, respectively. For the random-forest-based substructure fingerprint prediction model, 30 molecules were identified and removed using PCA. Our optimized random-forest-based substructure fingerprint model demonstrated correlation coefficients of 0.9796 and 0.9531, RMSEs of 0.2971 and 0.2288, and MAEs of 0.1683 and 0.2104 for the training and test sets of 163 and 51 molecules, respectively. For the random-forest-based 1D and 2D molecular descriptor prediction models, we identified and removed 40 molecules using PCA. Our optimized random-forest-based 1D and 2D molecular descriptor models demonstrated correlation coefficients of 0.9852 and 0.8803, RMSEs of 0.2452 and 0.372, and MAEs of 0.1874 and 0.3068 for the training set of 167 molecules and the test set of 52 molecules, respectively (Table 1, Figs. 4 and 5).

**Table 1:** Validation matrices of all the generated multifeature ML-QSAR prediction models.

Prediction Model	PubChemFP prediction model (Training)	PubChemFP prediction model (Test)	SubFP prediction model (Training)	SubFP prediction model (Test)	1D and 2D mol. desc prediction model (Training)	1D and 2D mol. desc prediction model (Test)
Correlation coefficient	0.9863	0.9397	0.9796	0.9531	0.9852	0.8803
MAE	0.1645	0.2389	0.1683	0.2104	0.1874	0.3068
RMSE	0.212	0.2971	0.2288	0.3023	0.2452	0.372
RAE	16.9107%	29.3921%	18.3017%	25.0749%	19.691%	45.1193%
RRSE	19.1019%	33.0025%	21.6219%	31.3838%	23.0722%	47.8062%
Total Number of Instances	168	56	163	51	167	52

### Interpretation of the ML-QSAR model

**Figure 6:**
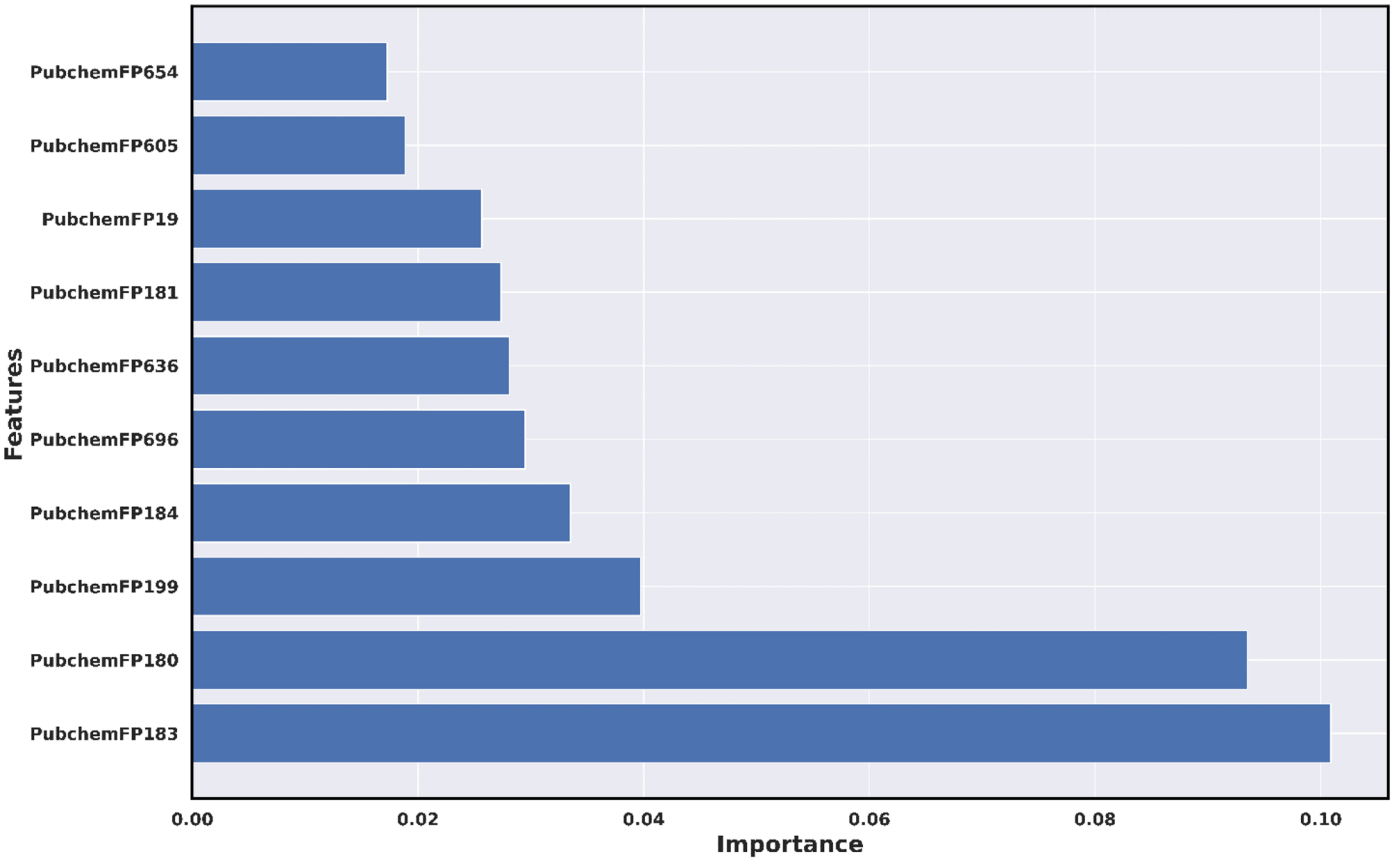
VIP plot analysis of ten optimal features of the PubChem prediction model.

**Figure 7:**
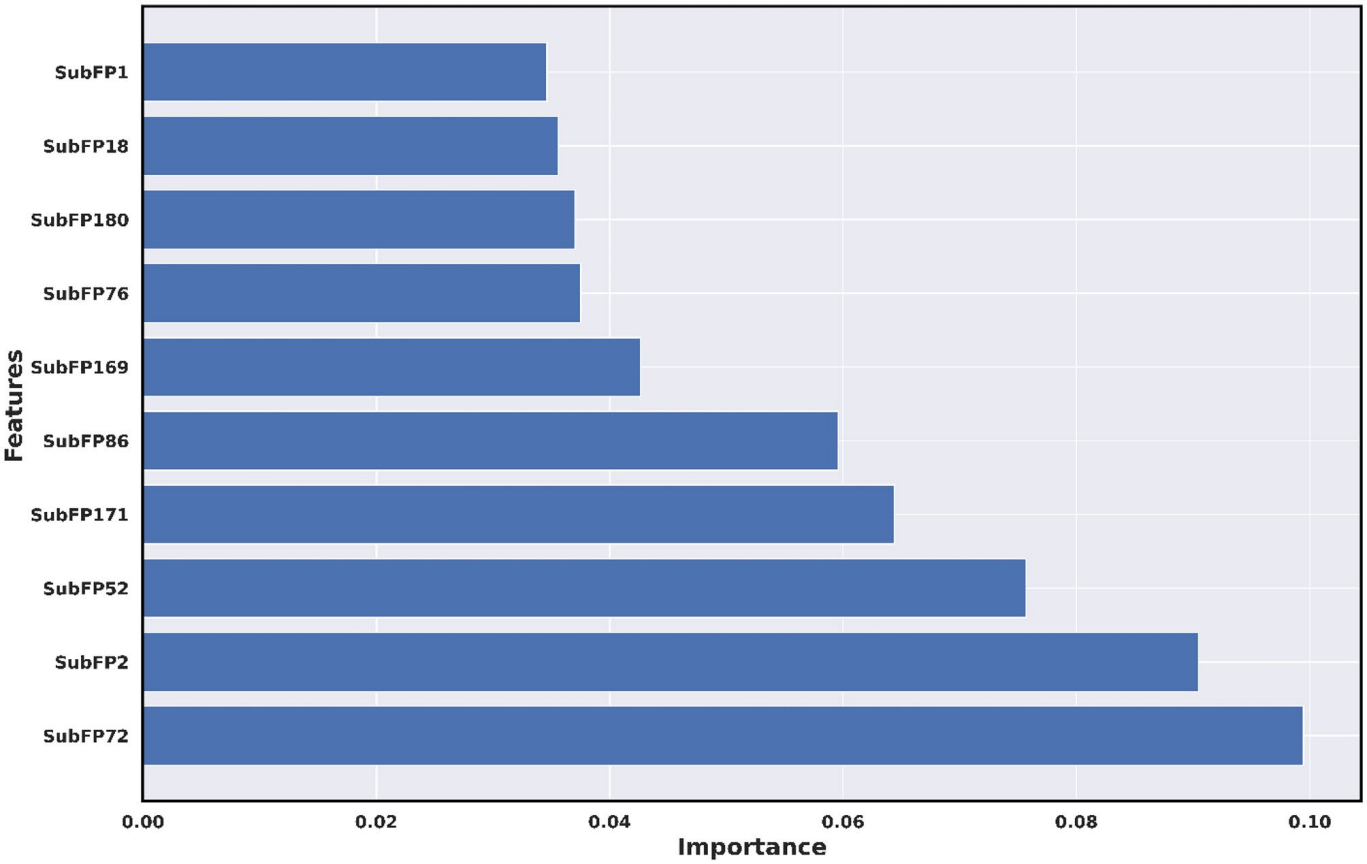
VIP plot analysis of ten optimal features of the substructure prediction model.

**Figure 8:**
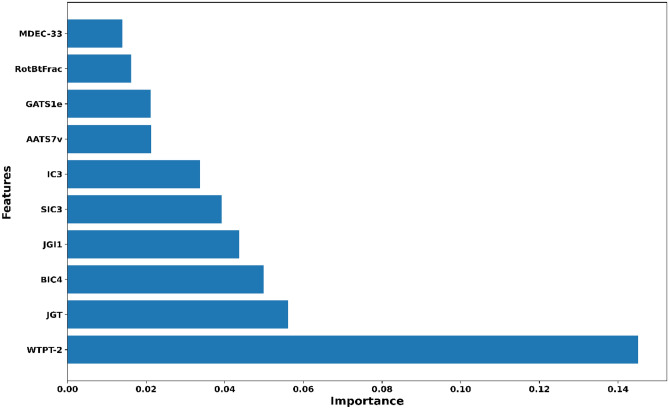
VIP plot analysis of ten optimal features of the 1D and 2D molecular descriptor prediction model.

By implementing variance importance plots (VIP), we extracted the ten optimal molecular features for each ML-QSAR prediction model. PubChem prediction model, we extracted PubchemFP654, PubchemFP605, PubchemFP19, PubchemFP181, PubchemFP636, PubchemFP696, PubchemFP184, PubchemFP199, PubchemFP180, and PubchemFP183 as the ten optimal were features for the model through VIP prediction model. For the random-forest-based substructure prediction model, we extracted SubFP1**,** SubFP18, SubFP180, SubFP76, SubFP169, SubFP86, SubFP171, SubFP52, SubFP2, and SubFP72 as the ten optimal molecular features for model generation using VIP plot analysis. Regarding the random-forest-based 1D and 2D molecular descriptor prediction model, we extracted MDEC-33, RotBtFrac, GATS1e, AATS7v, IC3, SIC3, JGI1, BIC4, JGT, and WTPT-2 as the ten optimal molecular features for the generation of the model through VIP plot analysis. Following VIP plot analysis, we implemented a correlation matrix for the ten optimal molecular features for each ML-QSAR prediction model. Furthermore, we determined that the extracted ten optimal features extracted from each ML-QSAR prediction model were correlated. All key features extracted through the VIP plot analysis were significantly involved in the inhibition mechanism of amide-based small molecules against MAO-B (Table 2, Figs. 6, 7, 8, and Supplementary Figures S1‒S3).

**Table 2:** Molecular feature interpretation of all generated ML-QSAR prediction models.

FINGERPRINTS	INTERPRETATION	FINGERPRINTS	INTERPRETATION	MOLECULAR DESCRIPTORS	INTERPRETATION
PubchemFP654	N–C–N–C–C	SubFP1	Primary carbon	MDEC-33	Molecular distance edge
PubchemFP605	O=C–C–O–C	SubFP18	Alkylarylether	RotBtFrac	Rotatable bonds count
PubchemFP19	> = 2 O	SubFP180	Hetero N basic no H	GATS1e	Autocorrelation
PubchemFP181	> = 1 saturated or aromatic heteroatom-containing ring size 6	SubFP76	Enamine	AATS7v	Autocorrelation
PubchemFP636	C–N–C–N–C	SubFP169	Phenol	IC3	Information content
PubchemFP696	C–C–C–C–C–C–C–C	SubFP86	Lactone	SIC3	Information content
PubchemFP184	> = 1 unsaturated non-aromatic heteroatom-containing ring size 6	SubFP171	Arylchloride	JGI1	Topological charge
PubchemFP199	> = 4 any ring size 6	SubFP52	Imine	BIC4	Information content
PubchemFP180	> = 1 saturated or aromatic nitrogen-containing ring size 6	SubFP2	Secondary carbon	JGT	Topological charge
PubchemFP183	> = 1 unsaturated non-aromatic nitrogen-containing ring size 6	SubFP72	Enol	WTPT-2	Weighted path

**Figure 9:**
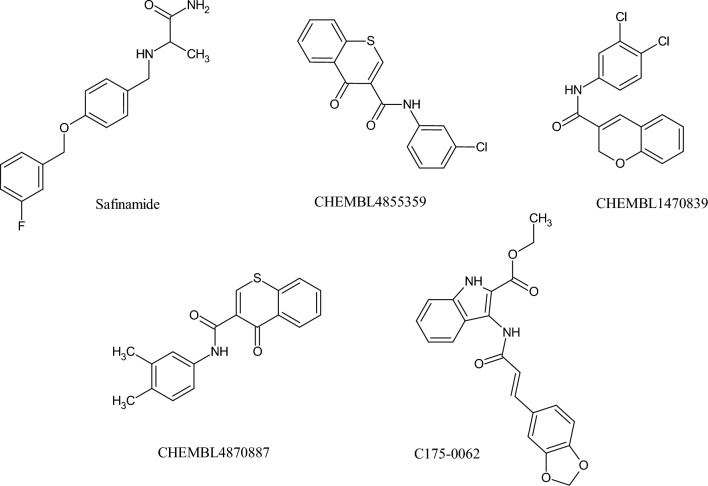
Chemical structures of previously known amide-based MAO-B inhibitors along with newly discovered molecule C175-0062.

**Figure 10:**
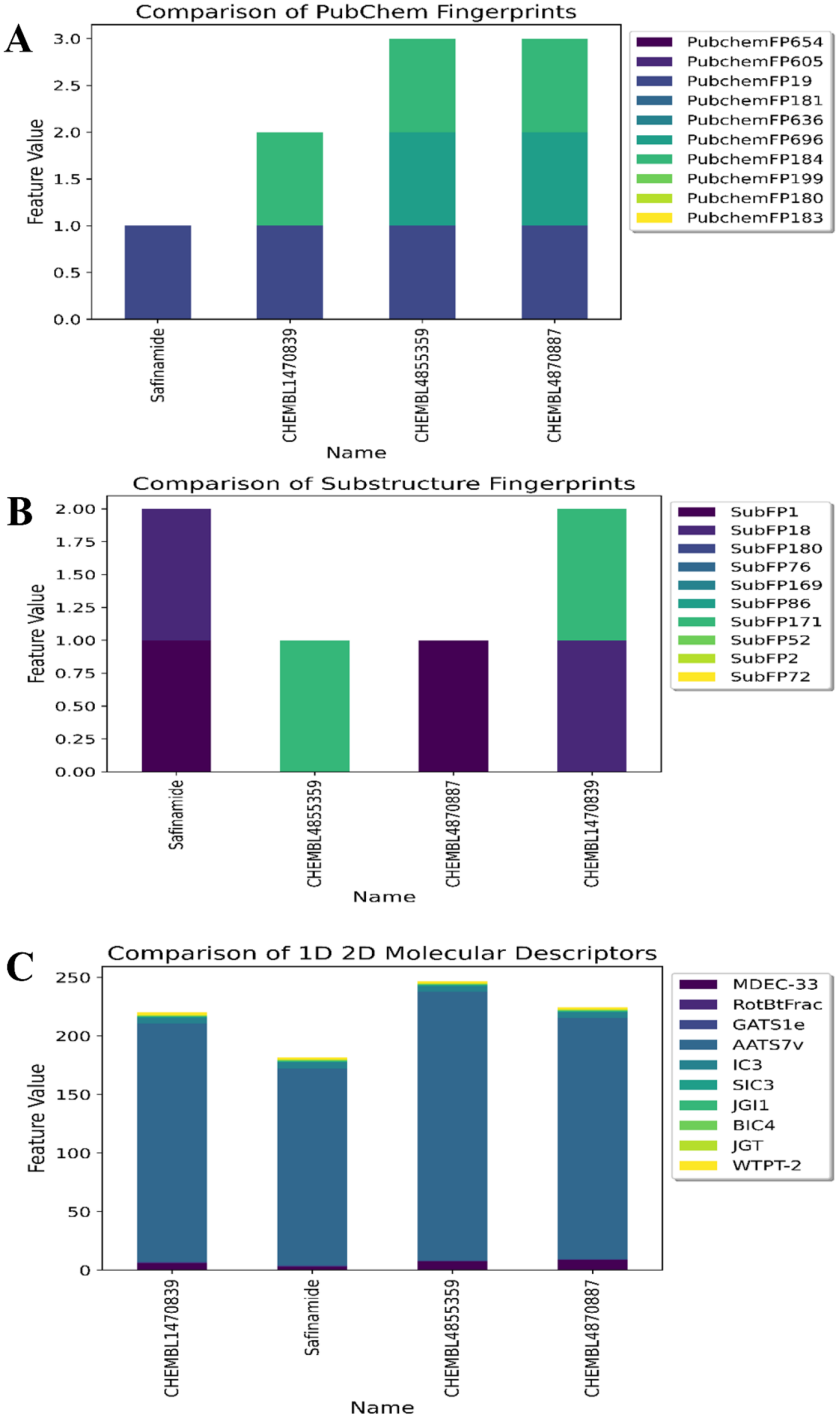
Structural analysis of optimal active molecules of the QSAR dataset in contradiction with VIP plot extracted features. (**A**) ML-QSAR PubChem fingerprint prediction model; (**B**) ML-QSAR substructure fingerprint prediction model; (**C**) ML-QSAR 1D 2D molecular descriptor prediction model.

We further investigated the structure of the top bioactive molecules in our dataset, along with the FDA-approved MAO-B inhibitor safinamide. The molecular signatures of three bioactive molecules in our dataset, CHEMBL4855359, CHEMBL1470839, and CHEMBL4870887, with experimental pIC_50_ values of 8.82, 8.5, and 8.475, respectively, were compared with those of safinamide, which demonstrated an experimental pIC_50_ of 7.10. For the random-forest-based PubChem prediction model, CHEMBL4855359, CHEMBL1470839, CHEMBL4870887, and safinamide predicted pIC_50_ values of 8.136, 7.98, 8.138, and 6.93, respectively. Furthermore, the structural analysis of all four molecules demonstrated the presence of PubChemFP19. CHEMBL4855359, CHEMBL1470839, and CHEMBL4870887 demonstrated the presence of PubChemFP184. CHEMBL4855359 and CHEMBL4870887 contained PubChemFP696. Therefore, among the 10 optimal PubChem fingerprints extracted through VIP plot analysis, PubChemFPs 19 (presence of higher oxygen count), 184 (> = 1 unsaturated non-aromatic heteroatom-containing ring size 6), and 696 (presence of higher carbon count of at least 8) were mostly responsible for high inhibition activity against MAO-B. Similarly, in the random-forest-based substructure prediction model, CHEMBL4855359, CHEMBL1470839, CHEMBL4870887, and safinamide had pIC_50_ values of 8.26, 7.83, 8.58, and 6.927, respectively. Safinamide and CHEMBL4870887 both demonstrated the presence of SubFP18, whereas safinamide and CHEMBL1470839 demonstrated the presence of SubFP180. Furthermore, CHEMBL4855359 and CHEMBL1470839 contained SubFP171. This suggests that among the 10 best substructure fingerprints extracted through VIP plot analysis, SubFPs 18 (alkylarylether), 180 (Hetero N basic no H), and 171 (arylchloride) were mostly responsible for the high inhibitory activity against MAO-B. Finally, for the random-forest-based 1D and 2D prediction model, CHEMBL4855359, CHEMBL1470839, CHEMBL4870887, and safinamide demonstrated predicted pIC_50_ values of 8.56, 8.24, 8.83, and 6.46 respectively. The ranges of the molecular descriptor values of WTPT-2, JGT, BIC4, JGI1, SIC3, and IC3 were nearly identical for all four molecules used for comparative analysis. The molecular descriptor MDEC-33 was shown to be in the range 3–8.8, RotBtFrac was shown to be in the range 0.1–0.4, GATS1e was shown to be in the range 0.57–0.71, and AATS7v was shown to be in the range 168–229, respectively, for all molecules. Based on the statistical and validation metrics, our three ML-QSAR multi-feature prediction models were highly robust and interpretable for screening large databases for anti-PD drug discovery concerning MAO-B inhibition (Figs. 9 and 10A–C).

### Web application deployment for the generated ML-QSAR models

We developed a Python-based web application, MAO-B-pred (https://mao-b-pred.streamlit.app/) using the Streamlit library. This application leverages previously established molecular fingerprints and molecular descriptor-based small-molecule prediction models for MAO-B. To create the web application, various Python libraries were used, including scikit-learn v1.2.0. (https://scikit-learn.org/stable/whats_new/v1.2.html), pandas (https://pandas.pydata.org/docs/user_guide/10min.html), subprocesses, os, base64, and pickles (https://www.askpython.com/python-modules/install-pickle-python). MAO-B-pred operates by considering the SMILES representations of multiple molecules along with their corresponding names or IDs provided by the user within a text file. Upon uploading the text file containing the molecular information, the application predicted the inhibitory activity (pIC_50_) of the loaded molecules against MAO-B. This application uses PaDEL software along with established fingerprint- and descriptor-based small-molecule prediction random forest models to calculate the significant molecular fingerprints and molecular descriptors for the loaded molecules. Subsequently, the predicted activity is displayed in the form of pIC_50_ values along with their respective molecular names. Users can download the activity values and molecule names in the CSV format directly from the application. The complete source code for the MAO-B-pred is openly accessible at https://github.com/RatulChemoinformatics/MAO-B. To use the application on personal workstations, users must have the Anaconda Navigator interface installed on their systems, along with StreamLit and other necessary package dependencies. The installation process is detailed in the readership file available in the GitHub repository. By following these instructions, users can accurately predict the molecular activity of MAO-B using the MAO-B-pred application.

### Virtual screening for anti-PD drug discovery

Using the developed ML-QSAR-based web application, we screened an oxidoreductase compound library on the ChemDiv website (https://www.chemdiv.com/catalog/focused-and-targeted-libraries/privileged-fragments-annotated-library/). A total of 8812 molecules were initially screened using the Chembioserver 2.0 web platform (https://chembioserver.vi-seem.eu/simple_search.php) based on three drug-likeness rules: Lipinski’s rule of five, Veber rules, and Ghose filters. Following the initial drug-likeness screening, we further screened the molecules passing through the drug-likeness filters by subjecting them to our ML-based web application MAO-B-pred. We screened the molecules individually to predict their bioactivity against MAO-B using three multi-feature-based prediction models: a PubChem fingerprint-based prediction model, a substructure fingerprint-based prediction model, and a 1D and 2D molecular descriptor-based prediction model. We further identified 1946 molecules that demonstrated a predicted pIC_50_ value of ≥ 6 (IC_50_: 100–1000 nM) for all ML-QSAR prediction models and subjected them to molecular docking analysis.

### Molecular docking and structural analysis based on ML-QSAR models

**Figure 11:**
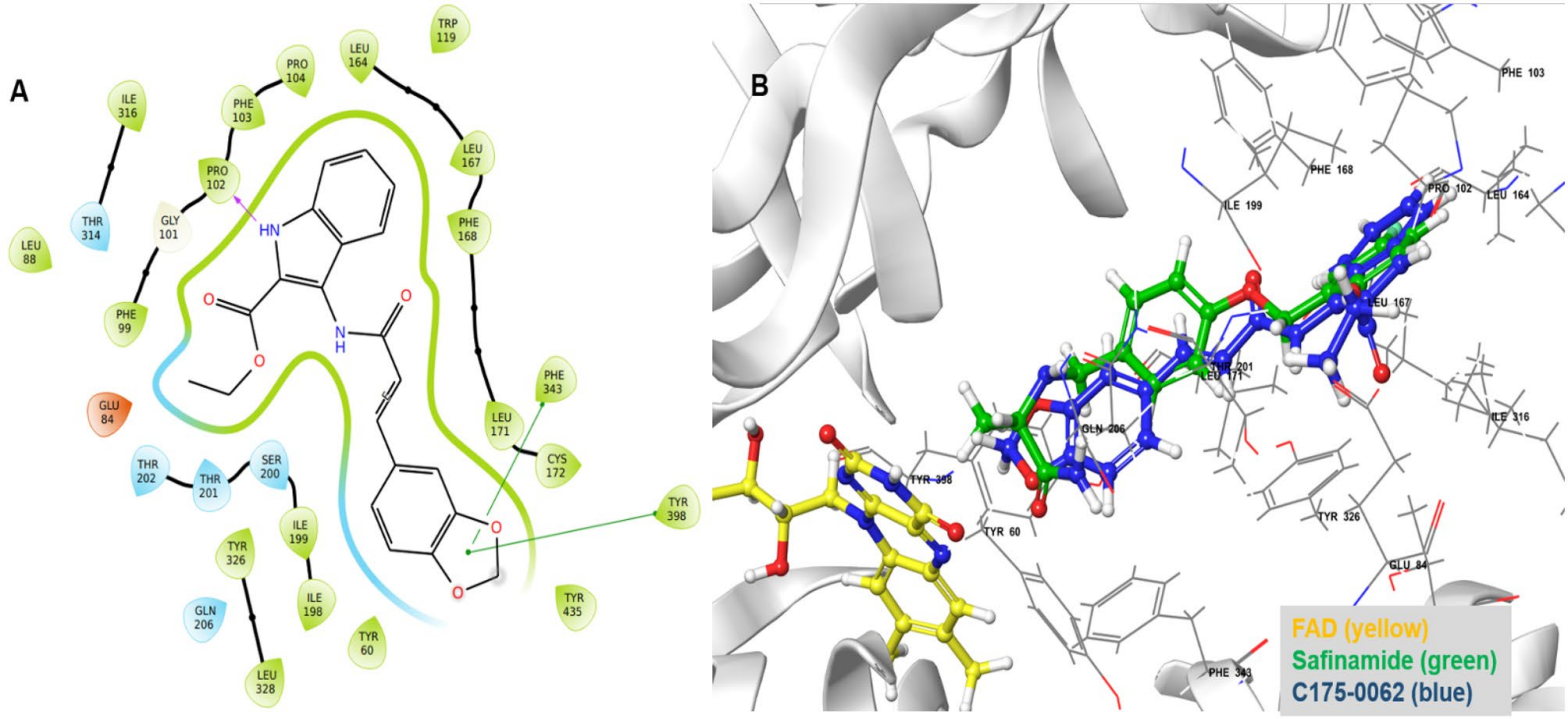
2D (**A**) and 3D (**B**) interactions of **C175-0062** with the MAO-B binding pocket.

**Figure 12:**
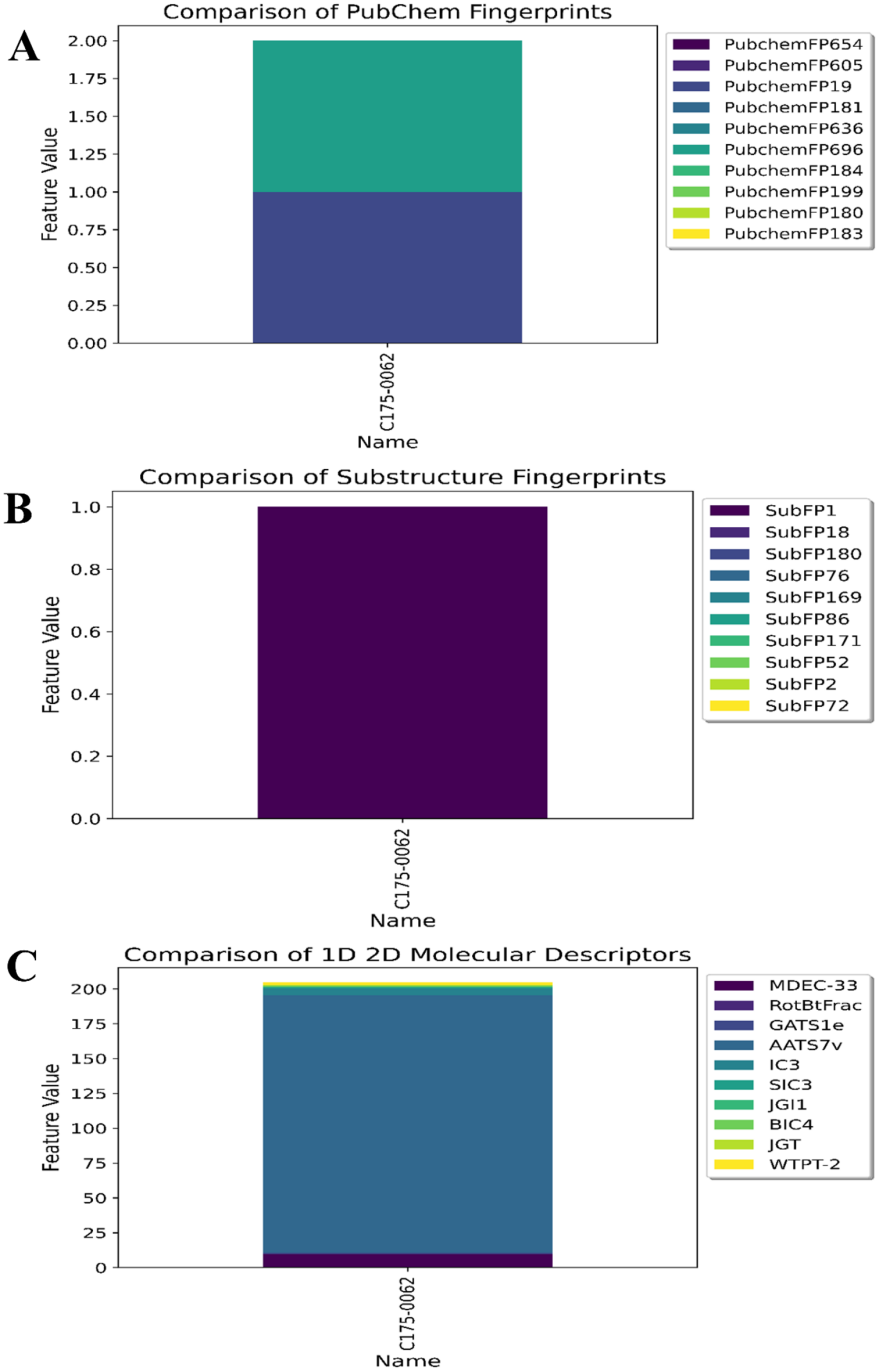
Structural analysis of C175-0062 in contradiction with VIP plot extracted features. (**A**) ML-QSAR PubChem fingerprint prediction model; (**B**) ML-QSAR substructure fingerprint prediction model; (**C**) ML-QSAR 1D 2D molecular descriptor prediction model.

An oxidoreductase library consisting of 1986 molecules with predicted pIC_50_ values of 6 and above was used for ligand-based virtual screening (https://www.chemdiv.com/catalog/focused-and-targeted-libraries/privileged-fragments-annotated-library/). Subsequently, these molecules were screened using high-throughput virtual screen (HTVS) (> 8 kcal/mol), standard precision (SP) (> 8 kcal/mol), and extra precision (XP) methods. Docking analyses were conducted to gain a comprehensive understanding of the binding mechanisms of the compound and evaluate the impact of structural modifications on its inhibitory activity against hMAO-B. The X-ray crystal structure of hMAO-B (PDB ID: 2V5Z) was obtained. As indicated in Table S1, all the evaluated compounds exhibited substantial docking scores for hMAO-B. Among the 176 hits, the docking scores ranged 0.54 to–13.499 kcal/mol in XP mode. Notably, compound C175-0062 displayed a docking score of -13.499 kcal/mol, comparable to safinamide's score (-13.40 kcal/mol) against MAO-B. The binding interaction between C175-0062 and safinamide in the hMAO-B pocket is illustrated in Fig. 11B, which reveals a similar orientation for both the native ligand and C175-0062. An amide is close to binding to FAD in the case of a native ligand, whereas methylenedioxy is close to binding to FAD in our lead molecule. The amide of the lead molecule is bound in close proximity to the gate residue of the amino acid, Ile99. Docking poses on hMAO-B revealed that the secondary nitrogen (–NH–) atom of the indole group in the structure formed hydrogen bonds with Pro102 (2.22 Å). Additionally, the benzo-1, 3-dioxole group of C175-0062 engaged in bipartite pi-pi stacking interactions with Tyr398 and Phe343. Thus, the presence of a hydrogen bond donor group, such as the NH group, at this location may contribute favorably to the biological activity. Hydrophobic interactions surrounded by the amino acids (Fig. 11A) Tyr60, Leu88, Phe99, F103, Pro104, Trp119, Leu164, Leu167, F168, Leu171, C172, I198, I199, L328, and Y326 in MAO-B were also favorable. Additionally, polar interactions were observed with Ser200, Thr201, Thr202, Gln206, and Thr314 at the active sites of the enzyme. Furthermore, we analyzed the key structural features of C175-0062 and aligned them against the extracted key features of each ML-QSAR model previously studied using VIP plot analysis. Concerning the PubChem fingerprints and substructure fingerprint prediction model, molecule C175-0062 displayed significant molecular signatures PubChemFPs 696 and 19, along with SubFP1, which are key molecular features involved in regulating the inhibitory activity against MAO-B. For the 1D 2D molecular descriptor prediction model, molecule C175-0062 displayed significantly higher values for the molecular descriptors AATS7v, MDEC-33, and IC3. These findings emphasize that the molecule C175-0062 inherited key structural characteristics involved in the higher inhibition activity of previously known MAO-B amide-based inhibitors (Figs. 11 and 12A–C).

### MD study

**Figure 13:**
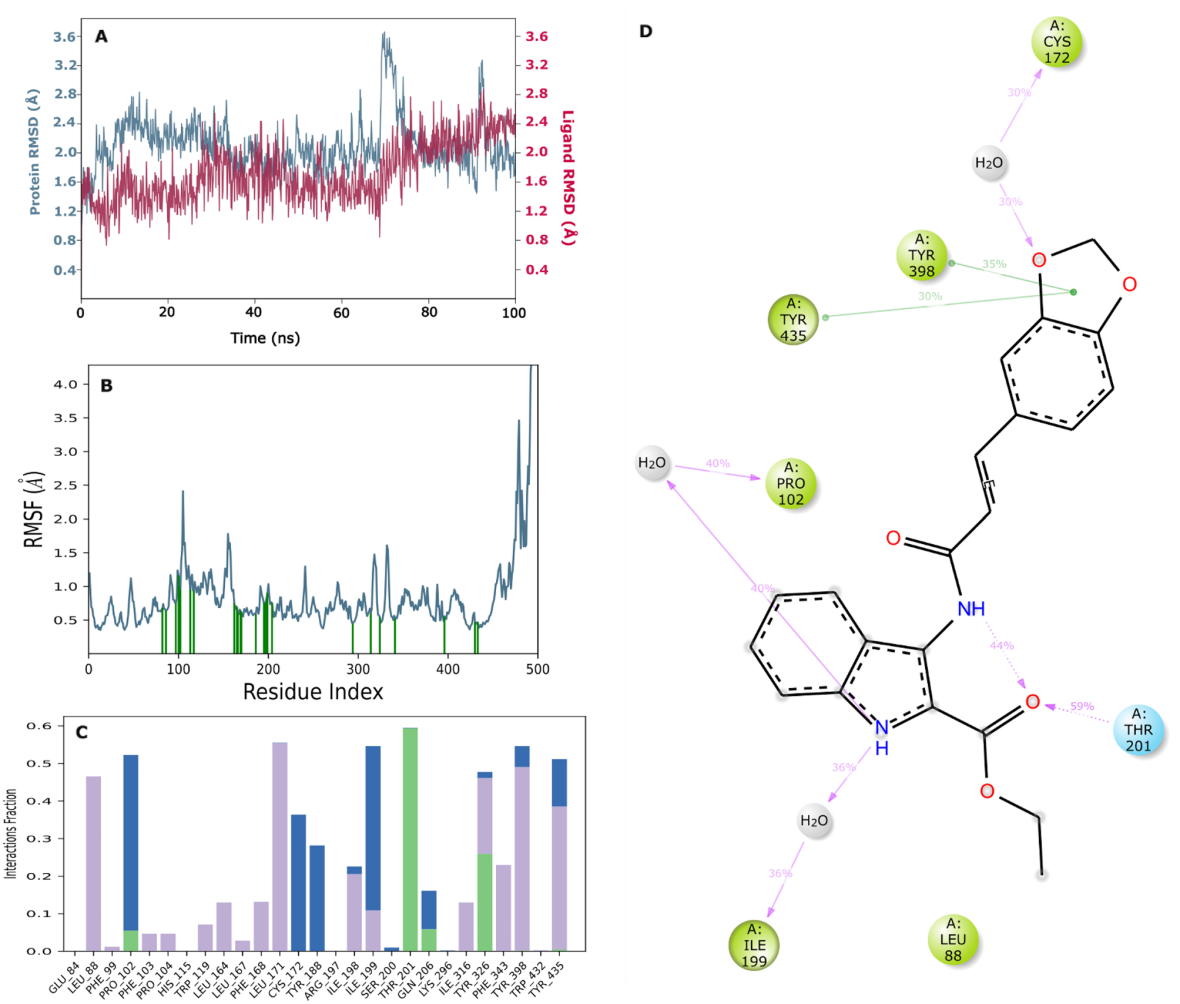
Analysis of the C175-0062 -MAO-B complex using MD simulation. (**A**) RMSD (protein RMSD is shown in blue, and RMSD of C175-0062 is shown in red). (**B**) Individual amino acid RMSF for proteins. (**C**) Analysis of protein–ligand contacts of MD trajectory. (**D**) 2D Interaction diagram.

To investigate the stability and flexibility of the complex, MD simulations were performed on the docked complex of C175-0062 at the binding site of the MAO-B protein in biological environments. MD trajectories were analyzed to calculate the root-mean-square deviation (RMSD), root-mean-square fluctuation (RMSF), and protein–ligand interactions. The figure shows various analyses of the MD trajectory data for the C175-0062-MAO-B complex (Fig. 13).

### RMSD

The RMSD plot indicated a stable ligand–protein complex throughout the simulation, with RMSD values ranging 0.9–3.6 Å for the Cα atoms of the protein in complex with C175-0062. The ligand RMSD ranged 0.7–2.8 Å with regard to the protein, with average RMSD values of 2.10 Å for the protein and 1.720 Å for the ligand. The RMSD of the protein remained constant throughout the simulation, with a slight variation observed at 68–72 ns. In contrast, the ligand RMSD exhibited minor fluctuations until 70 ns, after which it increased and remained constant until the end of the simulation (Fig. 13A). The RMSD values of the protein Cα atoms, derived from the simulated trajectory of the protein–ligand complex, indicated the stability of the complex in a dynamic environment. A higher RMSD value for protein Cα atoms suggests unfolding, whereas a lower value indicates compactness. The modest variation in the backbone RMSD further supported the equilibration of the protein–ligand complex. The difference between the highest and lowest RMSD values signified the backbone deviation. In summary, the overall RMSD of the C175-0062-MAO-B complex is stable and consistent in a dynamic environment.

### RMSF

Throughout the simulation, the flexibility of the protein system was assessed by calculating the RMSF of individual amino acid residues. The RMSF plot revealed higher fluctuations in N-terminal residues. It was observed that during the simulation, compound C175-0062 interacted with 27 amino acids of MAO-B, namely Pro102 (1.165 Å), Phe103 (1.002 Å), His115 (0.95 Å), Pro104 (0.94 Å), Trp119 (0.935 Å), Thr201 (0.904 Å), Ser200 (0.824 Å), Arg197 (0.76 Å), Gln206 (0.756 Å), Ile199 (0.755 Å), Leu164 (0.746 Å), Phe99 (0.741 Å), Leu167 (0.693 Å), Ile198 (0.691 Å), Glu84 (0.679 Å), Leu171 (0.636 Å), Leu88 (0.632 Å), Cys172 (0.61 Å), Phe168 (0.601 Å), Ile316 (0.597 Å), Tyr188 (0.582 Å), Tyr398 (0.556 Å), Tyr326 (0.542 Å), Phe343 (0.521 Å), Trp432 (0.468 Å), Tyr435 (0.465 Å), and Lys296 (0.445 Å). All these interacting residues displayed RMSF values in the range 0.44–1.16 Å (Fig. 13B). Individual amino acid residues of the protein–ligand complex play a crucial role in maintaining the stability of dynamic processes. The RMSF parameter, derived from MD simulation trajectories, quantifies the variation in specific amino acids from the reference or native structure. The RMSF plot facilitates understanding of the residual vibrations in the C175-0062-MAO-B complex. The analysis indicated minimal conformational changes in the amino acids in the binding cavity of MAO-B, particularly those interacting with the reported lead compound, as highlighted by the green vertical bar in the RMSF plot. This observation suggests that the active site and main chain residues experienced only slight fluctuations, indicating firm binding of the lead compound within the binding pocket of the target protein with minor conformational changes.

### Protein ligand contact analysis

Protein–ligand contact analysis based on ligand-mediated two-dimensional interactions demonstrated that the hydrophobic amino acids Tyr398 and Tyr435 engage in significant bipartite π–π stacking with the benzo-dioxole moiety of compound C175-0062, accounting for 35% and 30% of the simulation time, respectively (Fig. 13C). In addition, the carbonyl group of the amide linker participated in hydrogen bonding interactions for 59% of the simulation time. Amino acids Ile199 and Pro102 formed dipartite hydrogen bonds with the nitrogen atom of the indole group, contributing 36% and 40%, respectively. Furthermore, Cys172 exhibited a 30% interaction through a water-mediated hydrogen bond with the oxygen atom of benzo-dioxole. The Fig. 13D illustrates the binding interactions between C175-0062 and active site amino acid residues within the binding pocket of the MAO-B protein. MD simulations revealed that the predominant interactions involved hydrophobic interactions, hydrogen bonds, and polar interactions (water-mediated hydrogen bonding). According to protein–ligand contact analysis, Glu84, Pro102, Leu171, Cys172, Ile199, Thr201, Thr326, Tyr398, and Tyr435 exhibited significant contact with C175-0062. Comparing the 2-D interaction of the ligand during docking with that after simulation showed similar interactions. The simulation results for compound C175-0062 indicated comparable hydrophobic and hydrogen bonding interactions with Leu171 and Gln201, respectively, suggesting its potential as an inhibitor of hMAO-B.

### PCA

**Figure 14:**
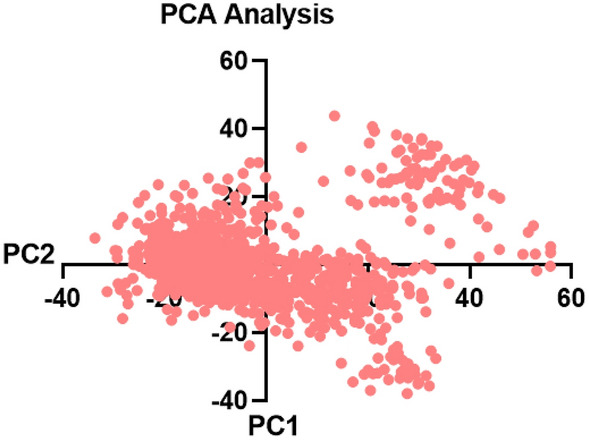
PCA of C175-0062-hMAO-B protein–ligand complex.

The large-scale collective motions of the protein in protein–ligand complexes on the trajectories produced by simulations were investigated, and the conformational distribution during the simulation period was understood through the application of the PCA approach. To anticipate the dynamic behavior of a protein88, the Essential dynamics (ED) analysis script of the Desmond program (trj_essential_dynamics.py) was utilized through a command line. The primary constituents of the Cα atoms in proteins are computed by this script. Through simulations, a uniform distribution of conformations was observed in the complex through phase–space projection of protein motion along PC1 and PC2. The aforementioned RMSD, RMSF, and PCA values, which were obtained from the MD simulation trajectories, illustrated the stability of the C175-0062-hMAO-B protein–ligand complex in dynamic states (Fig. 14).

### Molecular mechanics/generalized born surface area (MM-GBSA)

Based on its molecular MD simulation frames, the free binding energy of the ideal molecule, C175-0062, which had the highest docking energy and predicted activity value, was estimated. Over the 10 to 100 ns MD snapshot, the total average energies for ΔG Bind, ΔG Bind H-bond, ΔG Bind Lipo, and ΔG Bind vdW were determined to be − 150.35, − 11.50, − 33.27, and − 120.01, respectively. Based on the study of these energies, Table 3 shows that ΔG Bind and ΔG Bind Lipo had the biggest effects on the average binding energy for all interactions.

**Table 3:** Free binding energies of the molecule C175-0062 shown through MM-GBSA*.

MD (ns)	ΔG Bind	ΔG Bind H–bond	ΔG Bind Lipo	ΔG Bind vdW
10	− 145.07	− 11.36	− 31.14	− 122.58
20	− 156.14	− 12.17	− 33.36	− 121.37
30	− 147.79	− 11.78	− 32.25	− 124.80
40	− 152.29	− 11.67	− 33.56	− 116.25
50	− 153.08	− 11.63	− 35.99	− 118.80
60	− 158.35	− 11.91	− 33.14	− 122.30
70	− 154.31	− 11.50	− 33.06	− 118.39
80	− 152.88	− 11.12	− 34.33	− 121.24
90	− 152.27	− 10.96	− 33.51	− 124.09
100	− 131.27	− 10.93	− 32.37	− 110.33

*kcal/mol.

In particular, stable van der Waals contacts with amino acid residues were revealed by the ΔG Bind vdW values for C175-0062's interactions with protein complexes. The binding energies determined from the docking data and MM-GBSA computations, which were based on MD simulation trajectories, were consistent. Significantly, the molecule showed low free binding energy, indicating a high affinity for binding to the receptor. This suggests that C175-0062 has a strong affinity for MAO-B.

## Conclusion

Our study aimed to apply ligand-based drug design approaches to generate multi-feature ML-QSAR prediction models to unravel the structural features of MAO-B inhibitors behind their bioactivity mechanism. We developed three different molecular feature-based ML-QSAR models by implementing PubChem fingerprints, substructure fingerprints, and 1D and 2D molecular descriptors. The final PubChem fingerprint model, based on the random forest ML algorithm and 246 molecular features, showed correlation coefficients of 0.9863 and 0.9397, root mean square errors (RMSEs) of 0.212 and 0.2971, MAEs of 0.1645 and 0.2389 for the training and test sets of 168 and 56 molecules, respectively. The final substructure fingerprint model, based on a random forest ML algorithm and 56 significant features, achieved correlation coefficients of 0.9796 and 0.9531, RMSEs of 0.2288 and 0.3023, and MAEs of 0.1683 and 0.2104 for training and test sets of 163 and 51 molecules, respectively. The final model, based on a random forest ML algorithm and the implementation of 472 1D and 2D molecular descriptors, showed correlation coefficients of 0.9852 and 0.8803, RMSEs of 0.2452 and 0.372, and MAEs of 0.1874 and 0.3068 for training and test sets of 167 and 52 molecules, respectively. We implemented the ML-QSAR prediction model to develop a Python-based web application, MAO-B-pred (https://mao-b-pred.streamlit.app/), using the Streamlit library. Using drug-likeness filters coupled with the generated web application bioactivity prediction function, we further screened an oxidoreductase compound library of 8812 molecules and isolated 1986 best-performing molecules with predicted IC_50_ values ranging 100–1000 nM. We identified C175-0062, as the best-performing molecules among the 1986 previously identified molecules against MAO-B, through extensive structural analysis based on key molecular features extracted from ML-QSAR models, molecular docking, interaction analysis, and MD simulation studies. However, additional in vitro and in vivo experiments are required to verify the inhibitory potential of C175-0062 used in our investigation. Existing machine learning models deployed as single web applications have demonstrated their capacity to yield valuable insights into structurally significant residues of the MAO-B protein. These residues play a crucial role in small molecule binding and hold unexplored possibilities for the development of highly effective MAO-B inhibitors, which could be used in the treatment of PD.

## Materials and methods

### Chemical data curation and standardization

We curated specific original articles concerning MAO-B amide-based inhibitors with IC_50_ values from previous literature and the ChEMBL database^27,28^. The initial step was the curation of previously discovered MAO-B inhibitors along with their corresponding structures in the SMILES format and bioactivity (IC_50_: Half-maximal inhibitory concentration) from the curated articles. IC_50_ values were converted to nM. Duplicate molecules, molecules with missing values, or relationships with bioactivity were removed. The molecules were further classified based on their IC_50_ bioactivity ranges. Molecules with an IC_50_ range of 100–1000 nM were classified as active, 1000–10,000 nM as intermediate, and the remainder as inactive.

### Exploratory space analysis

The Anaconda navigator and RDkit packages were installed in a Google Collab cloud-based notebook for the chemical space analysis step^23,29,30^. The RDkit package (https://www.rdkit.org/docs/source/rdkit.Chem.Lipinski.html) was used to calculate Lipinski’s rule for five molecular descriptors: molecular weight, Log P, hydrogen bond acceptor, and hydrogen bond donor. The establishment of a set of guidelines for the assessment of the drug-likeness of compounds was pioneered by Christopher Lipinski, a renowned chemist affiliated with Pfizer. The drug-likeness of a compound is determined by its absorption, distribution, metabolism, and excretion (ADME) characteristics, which collectively form its pharmacokinetic profile. Lipinski analyzed the entirety of orally active medications approved by the FDA, to establish a set of criteria now referred to as the "Rule of five" or "Lipinski's rule.” According to Lipinski's rule, which is a key principle in drug design and development, the molecular weight of a compound should be less than 500 Da. The octanol–water partition coefficient (Log P) should be less than 5. The number of hydrogen bond donors should be less than five, and the number of hydrogen bond acceptors should be less than ten. Matplotlib and the seaborn library of Python were used to visualize and compare the chemical spaces of the active and inactive molecules in our dataset using Lipinski’s rule. To provide a more consistent distribution of IC_50_ data, the IC_50_ values of the compounds were transformed into a negative logarithmic scale (pIC_50_), denoted as − log_10_(IC_50_).

### Molecular feature exploration

We used the PaDELPy module (https://github.com/ecrl/padelpy) of PaDEL software to explore and calculate the three different molecular features of previously curated MAO-B inhibitors^31^. The three molecular features, PubChem fingerprints, substructure fingerprints, and 1D and 2D molecular descriptors, were calculated individually for each curated MAO-B antagonists^31–33^. PubChem fingerprints provide insights into the atomic indices of molecules, substructure fingerprints provide information concerning different functional groups and patterns of molecules, and 1D and 2D descriptors provide information regarding various physicochemical and bond properties of molecules. Following the calculation of the molecular features, we used a feature selection technique using the variance threshold method to remove features with low variance.

### Dataset division and ML-QSAR model generation

Following feature selection of the significant molecular features individually for all three different molecular features, a dataset division was performed to divide each molecular feature dataset into training and test sets. A random 80:20 split was used for the training and test sets. The training and testing sets of each molecular feature dataset were further subjected to machine learning algorithms to generate three robust ML-QSAR bioactivity prediction models that correlated the molecular features with a pIC_50_ value^34–37^.

In the current study, we used a random forest ML algorithm to generate each of our multi-diverse molecular feature-based ML-QSAR models (PubChem fingerprints, substructure fingerprints, and 1D and 2D molecular descriptors) to correlate classification-based molecular fingerprints and numerical value-assigned molecular descriptors with biological activity (pIC_50_)^38–40^. Simple decision tree predictors are gathered to form an ensemble in a supervised machine-learning process called random forest. To create a single model that is more stable and accurate than a collection of individual decision trees, which may not always produce accurate predictions, several decision trees must be integrated into the model. Notably, random forests have been attempted to address the overfitting problem exhibited by decision trees. Moreover, a bootstrap aggregation or bagging approach was used to train the random forests. In bagging, the training data subsets are randomly sampled (with replacement), a model is fitted to the updated training sets, and the predictions are aggregated. Therefore, we developed universal random forest-based nonlinear prediction (ML-QSAR) models for MAO-B inhibitors by implementing diverse molecular properties to predict the activity of any new molecule against MAO-B.

### Validation and interpretation of the ML-QSAR models

Using the scikit-learn package, we calculated different validation metrics such as the correlation coefficient, MAE, RMSE, relative absolute error (RAE), and root relative squared error (RRSE) for all three ML-QSAR prediction models^41–44^. In addition, we conducted a VIP analysis to gain insights into the ten optimal molecular properties of each molecular feature prediction model. Furthermore, a correlation matrix was plotted against all ten optimal molecular properties extracted through the VIP plot analysis to further analyze the degree of correlation among the features. PCA was performed to improve the prediction model metrics by detecting all the outliers observed in the initial model generation. PCA is a linear adjustment that decreases the number of dimensions and identifies the direction of data with the highest variance. Given its inherent characteristics, this approach is highly responsive to variables with varying value ranges, including outliers. One benefit is that it enables data to be visualized in a scatter plot with two or three dimensions, thereby facilitating the visual confirmation of identified outliers. Moreover, it offers a high level of clarity for understanding the response variables. PCA offers the additional benefit of compatibility with several distance measures, enabling the enhancement of outlier detection accuracy when used in conjunction with other approaches. All graphical interpretations and analyses were performed using Matplotlib and Seaborn libraries in Python.

### Identification of new inhibitors for MAO-B using drug-likeness filtering, predictive modeling, and a binding affinity-assisted structure-based virtual screening strategy

For the virtual screening strategy, we initially retrieved the oxidoreductase compound library consisting of 8812 molecules from the ChemDiv website (https://www.chemdiv.com/catalog/focused-and-targeted-libraries/privileged-fragments-annotated-library/) in SDF format (structure data files). The library was initially screened using the Chembioserver 2.0 web platform (https://chembioserver.vi-seem.eu/simple_search.php) based on three drug-likeness rules, namely, Lipinski’s rule of five, Veber rules, and Ghose filters. Christopher Lipinski, a distinguished chemist associated with the pharmaceutical company Pfizer, spearheaded the development of a framework for evaluating the drug-like properties of substances. The drug-likeness of a substance is assessed based on its ADME properties, which collectively contribute to its pharmacokinetic profile. Lipinski analyzed all orally active drugs approved by the FDA. The objective of this analysis was to construct a set of criteria commonly known as the "Rule of five" or "Lipinski's rule." Lipinski's rule, a fundamental guideline in the field of drug design and research, stipulates that the molecular weight of a compound must be less than 500 Da. It is recommended that the octanol–water partition coefficient (Log P) should be below 5. It is recommended that the number of hydrogen bond donors should be limited to a maximum of five, whereas the number of hydrogen bond acceptors should not exceed ten. The Ghose filter is used in pharmaceutical research to predict drug-likeness in silico. It has limitations such as a partition coefficient ranging − 0.4–5.6, molecular weight between 160 and 480, molar refractivity ranging 40–130, and a total atom count ranging 20–70 atoms. Veber's rule (VR) enhances bioavailability standards with criteria such having < 10 rotatable bonds and maximum allowable polar surface area.

Following the initial drug-likeness screening, we further screened the molecules passing through the drug-likeness filters by subjecting them to our ML-based web application, MAO-B-pred. We screened the molecules individually to predict their bioactivity against MAO-B using three multi-feature-based prediction models: a PubChem fingerprint-based prediction model, a substructure fingerprint-based prediction model, and a 1D 2D molecular descriptor-based prediction model. We further identified only molecules that demonstrated a predicted pIC_50_ value of 6 and above (IC_50_:100–1000 nM) and subjected them to molecular docking analysis.

To identify promising lead compounds, virtual screening was undertaken for molecules with predicted pIC_50_ value of 6 and above to evaluate their binding affinities to the active sites of the human MAO-B enzyme (PDB ID: 2V5Z). Active sites of the enzyme were identified using a structure-based, in-silico method. For a comprehensive examination of ligand-binding affinities, HTVS, SP, and XP docking techniques with a default force field were applied^45^.

Molecular docking used a three-step approach of preprocessing, optimization, and protein energy minimization using the Protein Preparation Wizard (PPW) software (Schrödinger Release 2022–4: Protein Preparation Wizard; Epik, Schrödinger, LLC, New York, NY, 2024) program to prepare protein crystal structures. Ligands were prepared using LigPrep (Schrödinger Release 2022–4: LigPrep; Epik, Schrödinger, LLC, New York, NY, 2024), ensuring appropriate assignment of protonation states at pH 7.4 ± 1.0 and atom types. The bond orders were ascribed to the structures, and hydrogen atoms were added. A grid with x-, y-, and z-coordinates corresponding to the binding pockets was generated at the site of a co-crystallized ligand using a receptor grid-generating tool. Molecules with the optimal docking scores were used for MD simulation analysis.

### MD

The MD simulations were conducted to analyze the lowest docking pose of the lead compound using the OPLS4 force field in conjunction with the Desmond package (version 7.2). The simulations were performed by Dell, Inc. Precision 7820 Tower running Ubuntu 22.04.1 LTS 64-bit, equipped with an Intel Xeon (R) Silver 4210R processor, and an NVIDIA Corporation GP104GL (RTX A 4000) graphics processing unit. Further details regarding the MD study, including the solvent simulation box shape, size, barometer, and thermostat parameters, as well as long- and short-range interaction calculations, can be found in previous studies, as the same settings were applied to the systems under examination. Throughout the 100 ns MD simulation, an analysis of RMSD, RMSF, and protein–ligand contact was carried out across all Cα atoms to evaluate domain correlations^19–21,46^.

### MMGBSA

In computational chemistry and molecular dynamics simulations, a computational technique known as MM/GBSA is utilized to determine the free energy of binding between a protein and a ligand. This method comprises MM computations, surface area estimates, and a GB continuum solvent model that describes the internal energy of the system. MM/GBSA was calculated from the MD trajectory (0–100 ns) using the thermal_mmgbsa. py script^46^.

### Supplementary Information

Supplementary Information.
