# Supplementary material for: Machine learning driven web-based app platform for the discovery of monoamine oxidase B inhibitors
Source: Sci Rep. 2024 Feb 28;14:4868. doi: 10.1038/s41598-024-55628-y (PMC10901862; doi:10.1038/s41598-024-55628-y)
Supplement: Supplementary file 1 — Supplementary Information. [file 41598_2024_55628_MOESM1_ESM.docx]

**Supporting Information**

**Machine Learning Driven Web-based App Platform for the Discovery of Monoamine Oxidase B Inhibitors**

Sunil Kumar^1#^, Ratul Bhowmik^2#^, Jong Min Oh^3^, Mohamed A. Abdelgawad^4^, Mohammed M. Ghoneim^5^, Rasha Hamed Al‑Serwi^6^, Hoon Kim^3^*, Bijo Mathew^1^*

^1^ Department of Pharmaceutical Chemistry, Amrita School of Pharmacy, Amrita Vishwa Vidyapeetham, AIMS Health Sciences Campus, Kochi, India

^2^ Department of Pharmaceutical Chemistry, School of Pharmaceutical Education and Research, Jamia Hamdard, New Delhi, India

^3^ Department of Pharmacy, and Research Institute of Life Pharmaceutical Sciences, Sunchon National University, Suncheon 57922, Republic of Korea

^4^ Department of pharmaceutical chemistry, college of pharmacy, Jouf University, Sakaka 72341, Aljouf , Saudi Arabia

^5^ Department of Pharmacy Practice, College of Pharmacy, AlMaarefa University, Ad Diriyah, Riyadh, 13713, Saudi Arabia

^6^ Department of Basic Dental Sciences, College of Dentistry, Princess Nourah bint Abdulrahman University, P.O. Box 84428, Riyadh 11671, Saudi Arabia

^#^These authors contributed equally.

^*^Corresponding authors:

E-mail address: bijomathew@aims.amrita.edu; [bijovilaventgu@gmail.com](mailto:bijovilaventgu@gmail.com) (B. Mathew)

[hoon@sunchon.ac.kr](mailto:hoon@sunchon.ac.kr) (H. Kim)

**Table S1.** Docking scores of oxidoreductase library.

| **Code** | **Smiles** | **Docking score (XP mode)** |
| --- | --- | --- |
| **C175-0062** | **CCOC(c([nH]c1c2cccc1)c2NC(/C=C/c(cc1)cc2c1OCO2)=O)=O** | **-13.499** |
| **Safinamide** | **O=C(N)[C@@H](NCc2ccc(OCc1cccc(F)c1)cc2)C** | **-13.400** |
| Y504-5817 | O=C(COC(CCC(N1)=CNC1=O)=O)Nc1cccc(Cl)c1 | -12.701 |
| Y510-7269 | CC(/C(/C1=O)=C\c(cc2)cc(OC)c2O)=NN1c1cc(C(O)=O)ccc1 | -12.233 |
| Y511-9975 | Cc(cc1)ccc1NC(COC(CCC(N1)=CNC1=O)=O)=O | -12.090 |
| 4331-0844 | CCCCOC(c(cc1)ccc1/N=N/C(C(C)=C(C(N1)=O)C#N)=C1O)=O | -11.801 |
| K088-1488 | COc(ccc(/C=C1/N=C(c2cccc(Cl)c2Cl)OC1=O)c1)c1OC | -11.151 |
| 4331-0843 | CCOC(c(cc1)ccc1/N=N/C(C(C)=C(C(N1)=O)C#N)=C1O)=O | -11.116 |
| Y511-9322 | O=C(COC(CCC(N1)=CNC1=O)=O)Nc1ccccc1 | -11.065 |
| K088-1558 | Cc(c(C(OC1=O)=N/C1=C/c(cc1)cc(OC)c1OC)ccc1)c1[N+]([O-])=O | -10.999 |
| 4111-1463 | CC(C=C(C)N(C1=O)/N=C/c(cc2)cc(OC)c2O)=C1C#N | -10.980 |
| D083-0037 | CCCC(N=C(Nc1cc(C(OCCC)=O)ccc1)N1)=CC1=O | -10.893 |
| 8016-6853 | CC(N1)=CC(/C(/OC2=O)=C\c3c[nH]c(cc4)c3cc4F)=C2C1=S | -10.824 |
| Y507-3071 | CCOC(c(cc1)ccc1N(C(C1)=O)N=C1C(F)(F)F)=O | -10.779 |
| D083-0075 | CCCC(N=C(Nc1cc(C(OC(C)C)=O)ccc1)N1)=CC1=O | -10.759 |
| Y505-7258 | C/C(/Nc1cc(-c2ccccc2)n[nH]1)=C(/C(C=C(C)O1)=O)\C1=O | -10.732 |
| Y510-7102 | CCOc(cc(/C=C(\C(C)=NN1c(cccc2)c2Cl)/C1=O)cc1)c1O | -10.640 |
| 8010-6197 | CCOc(cc(/C=C(/C(C=C(C)N1)=C2C1=S)\OC2=O)cc1)c1O | -10.557 |
| 2124-0401 | [O-][N+](c1cc(C(OC2=O)=N/C2=C/c(cc2)cc3c2OCO3)ccc1)=O | -10.513 |
| D083-0074 | CCCC(N=C(Nc1cc(C(OCC)=O)ccc1)N1)=CC1=O | -10.512 |
| 1907-0020 | COc1cccc(C=C2C(Nc3cccc(Cl)c3)=O)c1OC2=O | -10.480 |
| K088-1762 | COc(ccc(C(OC1=O)=N/C1=C/c(cc1)cc(Cl)c1Cl)c1)c1OC | -10.458 |
| P733-0283 | CCOC(C(C(OCc(cc1)ccc1Cl)=O)=C1N2CCCCC1)=CC2=O | -10.439 |
| 6049-1096 | Cc(c(NC(C(O1)=Cc(cccc2)c2C1=O)=O)ccc1)c1Cl | -10.403 |
| 2125-0496 | Cc(ccc(C(OC1=O)=N/C1=C/c(cc1)cc(OC)c1OC)c1)c1[N+]([O-])=O | -10.402 |
| 8018-6899 | CC(N1)=CC(/C(/OC2=O)=C/c3cn(C)c(cc4)c3cc4F)=C2C1=O | -10.373 |
| 2125-0489 | Cc(ccc(C(OC1=O)=N/C1=C/c(cc1)cc2c1OCO2)c1)c1[N+]([O-])=O | -10.324 |
| 6049-1111 | O=C(C(O1)=Cc(cccc2)c2C1=O)Nc(cc1)ccc1Cl | -10.252 |
| K784-7342 | CC(C(C(NCc(c(F)cc(F)c1)c1F)=O)=C(C)O1)=CC1=O | -10.207 |
| G265-0081 | CC(C)C(SC1=NC(COC(c2c(C)cccc2)=O)=C2)=NN1C2=O | -10.199 |
| 5079-1278 | CCOC(C(C1=O)=C(C)N/C1=C\c1c(C(F)(F)F)cccc1)=O | -10.178 |
| 6049-1542 | Cc1cccc(NC(C(O2)=Cc(cccc3)c3C2=O)=O)c1 | -10.176 |
| K088-1748 | COc(ccc(C(OC1=O)=N/C1=C/c1cccc(Cl)c1)c1)c1OC | -10.158 |
| 8015-5009 | Cc(c(Br)c12)ccc2N=C(c(cc2)cc(OC)c2OC)OC1=O | -10.089 |
| 6049-1234 | [O-][N+](c(cc(cc1)NC(C(O2)=Cc(cccc3)c3C2=O)=O)c1Cl)=O | -10.034 |
| G265-0420 | O=C(c1cc(F)ccc1)OCC(N=C1SC=CN11)=CC1=O | -10.031 |
| K088-1741 | COc(ccc(C(OC1=O)=N/C1=C/c(cc1)ccc1Cl)c1)c1OC | -10.021 |
| 1889-3508 | [O-][N+](c1cc(/C=C(/C=C(c2ccccc2)O2)\C2=O)ccc1)=O | -9.925 |
| K088-1780 | COc(ccc(C(OC1=O)=N/C1=C/c(cccc1)c1[N+]([O-])=O)c1)c1OC | -9.923 |
| D083-0039 | CCCCOC(c1cccc(NC(N2)=NC(CCC)=CC2=O)c1)=O | -9.917 |
| 8005-2356 | CC(N1)=CC(/C(/OC2=O)=C/c(cc3)ccc3OC)=C2C1=S | -9.888 |
| Y500-9063 | COc1cccc(C=C2C(Nc(cccc3)c3[N+]([O-])=O)=O)c1OC2=O | -9.868 |
| D731-0580 | CCOc(cccc1)c1NC(C(O1)=Cc(cccc2)c2C1=O)=O | -9.853 |
| G265-0393 | O=C(c(cccc1)c1F)OCC(N=C1SC=CN11)=CC1=O | -9.838 |
| 8013-2896 | CC(N1)=CC(/C(/OC2=O)=C/c(cc3OCOc3c3)c3Br)=C2C1=O | -9.832 |
| 8014-7428 | O=C1OC(c2ccccc2)=C/C1=N/c(cc1)ccc1Cl | -9.811 |
| G265-0504 | CC(SC1=NC(COC(c(cccc2)c2Cl)=O)=C2)=CN1C2=O | -9.805 |
| G265-0792 | CC(ON12)=CC2=NC(COC(c2cccc(F)c2)=O)=CC1=O | -9.796 |
| G265-0517 | CC(SC1=NC(COC(c(cccc2)c2F)=O)=C2)=CN1C2=O | -9.776 |
| 8018-7903 | COc(cc(cc1C=C2C(Nc3ccccc3)=O)Cl)c1OC2=O | -9.757 |
| G265-0147 | CCC(SC1=NC(COC(COc2c(C)cccc2)=O)=C2)=NN1C2=O | -9.756 |
| 4695-1829 | Cc(ccc(C(COC(C(C(C)=C1)=C(C)OC1=O)=O)=O)c1)c1[N+]([O-])=O | -9.755 |
| D731-0691 | O=C(C(O1)=Cc(cccc2)c2C1=O)Nc(cccc1)c1Cl | -9.734 |
| G265-0541 | CC(SC1=NC(COC(c2cccc(Cl)c2)=O)=C2)=CN1C2=O | -9.714 |
| G265-0747 | CC(ON12)=CC2=NC(COC(c(cc2)ccc2F)=O)=CC1=O | -9.687 |
| 3257-0488 | CC(C(C(OCC(c(cc1)ccc1[N+]([O-])=O)=O)=O)=C(C)O1)=CC1=O | -9.675 |
| 5522-0174 | CC(Oc(ccc(C(O1)=Nc(ccc(Br)c2)c2C1=O)c1)c1OC)=O | -9.654 |
| 4100-1558 | COC(c(cccc1)c1NC(/C=C/c(cc1)cc2c1OCO2)=O)=O | -9.651 |
| 8014-2546 | O=C(/C=C/c1ccccc1)Nc(cc1)cc(CO2)c1C2=O | -9.650 |
| G265-0375 | O=C(c(cc1)ccc1F)OCC(N=C1SC=CN11)=CC1=O | -9.646 |
| K074-6552 | COc(ccc(/C=C1/N=C(c(cc2)ccc2[N+]([O-])=O)OC1=O)c1)c1OC | -9.644 |
| G265-0145 | CCC(SC1=NC(COC(c(cccc2)c2F)=O)=C2)=NN1C2=O | -9.641 |
| 8004-2826 | CC(N1)=CC(/C(/OC2=O)=C/c(cc(cc3)[N+]([O-])=O)c3O)=C2C1=S | -9.627 |
| 2576-4094 | CC(C)(OC(C1=Cc2c[nH]c3c2cccc3)=O)OC1=O | -9.609 |
| G265-0765 | CC(ON12)=CC2=NC(COC(c(cccc2)c2F)=O)=CC1=O | -9.600 |
| G265-0466 | CC(N12)=CSC2=NC(COC(CSc2ccccc2)=O)=CC1=O | -9.551 |
| Y507-3141 | CCCC(CC1=O)=NN1c(cc1)ccc1C(OCC)=O | -9.540 |
| G265-0454 | CC(N12)=CSC2=NC(COC(c2cc(C)ccc2)=O)=CC1=O | -9.531 |
| K088-3119 | COc(ccc(/C=C1/N=C(c(cc(cc2)[N+]([O-])=O)c2Cl)OC1=O)c1)c1OC | -9.475 |
| F502-0113 | CCOC(C(C=C1C(Nc2cccc(F)c2)=O)=C(C)NC1=O)=O | -9.463 |
| G264-4022 | CC(ON12)=CC2=NC(COC(CNC(c2ccccc2)=O)=O)=CC1=O | -9.455 |
| G265-0083 | CC(C)C(SC1=NC(COC(c(cccc2)c2F)=O)=C2)=NN1C2=O | -9.444 |
| G265-0437 | CC(N12)=CSC2=NC(COC(c(cc2)ccc2F)=O)=CC1=O | -9.405 |
| G265-0192 | CC(C)CC(SC1=NC(COC(c2ccco2)=O)=C2)=NN1C2=O | -9.398 |
| 8016-3281 | COc(ccc(C(Nc(cc1)cc(C(F)(F)F)c1Cl)O1)c2C1=O)c2OC | -9.386 |
| G357-1578 | CC(Nc1cccc(C(OCC(N=C2SC=CN22)=CC2=O)=O)c1)=O | -9.376 |
| 6049-1419 | CC(c(cc1)ccc1NC(C(O1)=Cc(cccc2)c2C1=O)=O)=O | -9.364 |
| 8017-2701 | CC(N1)=CC(/C(/OC2=O)=C/c(cccc3)c3Cl)=C2C1=O | -9.337 |
| 8007-0589 | O=C(CCC(OCC(C(F)F)(F)F)=O)Nc(cc1)ccc1Cl | -9.337 |
| G265-0380 | O=C(c(cccc1)c1Cl)OCC(N=C1SC=CN11)=CC1=O | -9.330 |
| G265-0764 | CC(ON12)=CC2=NC(COC(c2cc(C)ccc2)=O)=CC1=O | -9.326 |
| 1636-0225 | CCOC(c1c(NC(C2=Cc(cccc3)c3OC2=O)=O)sc(C)c1C)=O | -9.289 |
| G265-0455 | CC(N12)=CSC2=NC(COC(c(cccc2)c2F)=O)=CC1=O | -9.283 |
| 5080-3400 | CCOC(C(C1=O)=C(C)N/C1=C/c1cccc(Br)c1)=O | -9.253 |
| G265-0482 | CC(N12)=CSC2=NC(COC(c2cc(F)ccc2)=O)=CC1=O | -9.248 |
| 8007-7032 | [O-][N+](c1ccc(/C=C(/C=C(c2ccccc2)O2)\C2=O)cc1)=O | -9.225 |
| 4338-0850 | CC(/C1=C\c(cc2)cc(OC)c2OC(c2cccs2)=O)=NOC1=O | -9.218 |
| 6049-1163 | [O-][N+](c(cc1)cc(NC(C(O2)=Cc(cccc3)c3C2=O)=O)c1Cl)=O | -9.173 |
| Y501-5848 | CC(C)n1ncc(/C=C/C(C(C(OC(C)=C2)=O)=C2O)=O)c1C | -9.160 |
| G265-0479 | CC(N12)=CSC2=NC(COC(c2cccc(Cl)c2)=O)=CC1=O | -9.121 |
| C202-3505 | CCOC(C(C=C1C(Nc(cccc2)c2F)=O)=C(C)NC1=O)=O | -9.114 |
| G265-0480 | CC(N12)=CSC2=NC(COC(c(cc2)cc3c2OCO3)=O)=CC1=O | -9.103 |
| G265-0392 | Cc1cccc(C(OCC(N=C2SC=CN22)=CC2=O)=O)c1 | -9.028 |
| 4031-0015 | CCOC(C(C1=O)=C(C)N/C1=C/c1cccc(Cl)c1)=O | -9.017 |
| 1565-0039 | COc(ccc(C(O1)=Nc(ccc(Cl)c2)c2C1=O)c1)c1OC | -9.000 |
| G265-0144 | CCC(SC1=NC(COC(c2cc(C)ccc2)=O)=C2)=NN1C2=O | -8.997 |
| G265-0453 | CC(N12)=CSC2=NC(COC(c2c(C)cccc2)=O)=CC1=O | -8.987 |
| G265-0410 | COc(cc1)ccc1C(OCC(N=C1SC=CN11)=CC1=O)=O | -8.951 |
| G265-0003 | CC(SC1=NC(COC(c(cc2)ccc2F)=O)=C2)=NN1C2=O | -8.939 |
| F502-0109 | CCOC(C(C=C1C(Nc2c(C)cccc2)=O)=C(C)NC1=O)=O | -8.910 |
| 4533-0193 | C/C(/C(C(OC(C)=C1)=O)=C1O)=N\NC(c(cc1)ccc1[N+]([O-])=O)=O | -8.865 |
| G265-0436 | CC(N12)=CSC2=NC(COC(c2ccccc2)=O)=CC1=O | -8.861 |
| G265-0432 | O=C(CCc1ccccc1)OCC(N=C1SC=CN11)=CC1=O | -8.846 |
| 8013-2853 | Cc1c(C#N)[nH]c(C)c1/C=C(/C(C=C(C)N1)=C2C1=S)\OC2=O | -8.846 |
| G265-0501 | CC(SC1=NC(COC(c2cccs2)=O)=C2)=CN1C2=O | -8.801 |
| Y200-6452 | O=C(C1=Cc(cccc2)c2OC1=O)Nc(cc1)cc2c1OCO2 | -8.797 |
| G265-0489 | CCOc(cc1)ccc1C(OCC(N=C1SC=C(C)N11)=CC1=O)=O | -8.783 |
| Y021-6568 | CCOC(C1=CNC(N(CC2)c3c2cccc3)=NC1=O)=O | -8.735 |
| G265-0316 | O=C(c1ccco1)OCC(N=C1SC(C2CC2)=NN11)=CC1=O | -8.730 |
| G265-0536 | CC(SC1=NC(COC(c2cc(OC)ccc2)=O)=C2)=CN1C2=O | -8.721 |
| G265-0019 | CC(SC1=NC(COC(c2c(C)cccc2)=O)=C2)=NN1C2=O | -8.719 |
| G265-0045 | CC(SC1=NC(COC(c2cccc(Cl)c2)=O)=C2)=NN1C2=O | -8.704 |
| G265-0048 | CC(SC1=NC(COC(c2cc(F)ccc2)=O)=C2)=NN1C2=O | -8.699 |
| K088-1550 | Cc(c(C(OC1=O)=N/C1=C/c(cc1)cc2c1OCO2)ccc1)c1[N+]([O-])=O | -8.666 |
| G265-0476 | CC(N12)=CSC2=NC(COC(c(cc2)cc(OC)c2OC)=O)=CC1=O | -8.647 |
| 7445-0001 | O=C1OC(c(cc2)ccc2Cl)=C/C1=C/c1cnccc1 | -8.645 |
| G265-0404 | O=C(CSc1ccccc1)OCC(N=C1SC=CN11)=CC1=O | -8.634 |
| G265-0438 | CC(N12)=CSC2=NC(COC(c(cc2)ccc2Cl)=O)=CC1=O | -8.604 |
| G265-0544 | CC(SC1=NC(COC(c2cc(F)ccc2)=O)=C2)=CN1C2=O | -8.589 |
| 8008-3367 | Cc(cc1)ccc1C(OC1=O)=C/C1=C/c1ncccc1 | -8.553 |
| 8005-2367 | CC(N1)=CC(/C(/OC2=O)=C/c(cc3)ccc3OC)=C2C1=O | -8.529 |
| G265-0500 | CC(SC1=NC(COC(c(cc2)ccc2Cl)=O)=C2)=CN1C2=O | -8.514 |
| G265-0751 | CC(ON12)=CC2=NC(COC(c2ccc(C)cc2)=O)=CC1=O | -8.507 |
| G265-0423 | O=C(Cc1ccccc1)OCC(N=C1SC=CN11)=CC1=O | -8.500 |
| G265-0439 | CC(N12)=CSC2=NC(COC(c2cccs2)=O)=CC1=O | -8.451 |
| 8011-7300 | CC(N1)=CC(/C(/OC2=O)=C/c(cc(cc3)[N+]([O-])=O)c3O)=C2C1=O | -8.431 |
| G265-0950 | Cc1cccc(C(OCC(N=C2SC(CCOC)=NN22)=CC2=O)=O)c1 | -8.404 |
| G265-0409 | COc(cccc1)c1C(OCC(N=C1SC=CN11)=CC1=O)=O | -8.392 |
| G265-0427 | CCOc(cc1)ccc1C(OCC(N=C1SC=CN11)=CC1=O)=O | -8.389 |
| G265-0002 | CC(SC1=NC(COC(c2ccccc2)=O)=C2)=NN1C2=O | -8.375 |
| G265-0068 | CC(C)C(SC1=NC(COC(c2ccco2)=O)=C2)=NN1C2=O | -8.324 |
| G265-0379 | Cc(cc1)ccc1C(OCC(N=C1SC=CN11)=CC1=O)=O | -8.302 |
| G265-0401 | O=C(COc(cc1)ccc1F)OCC(N=C1SC=CN11)=CC1=O | -8.290 |
| G265-0887 | CCCC(SC1=NC(COC(c2c(C)cccc2)=O)=C2)=NN1C2=O | -8.277 |
| G265-0889 | CCCC(SC1=NC(COC(c(cccc2)c2F)=O)=C2)=NN1C2=O | -8.242 |
| G265-0315 | O=C(c1cccs1)OCC(N=C1SC(C2CC2)=NN11)=CC1=O | -8.228 |
| G265-0442 | CC(N12)=CSC2=NC(COC(c(cccc2)c2Cl)=O)=CC1=O | -8.193 |
| G265-0377 | O=C(c1cccs1)OCC(N=C1SC=CN11)=CC1=O | -8.125 |
| P163-0131 | CCOC(c1cccc(NC(C(C(Nc2c3scc2)=O)=C3O)=O)c1)=O | -8.125 |
| G265-0004 | CC(SC1=NC(COC(c(cc2)ccc2Cl)=O)=C2)=NN1C2=O | -8.039 |
| 2103-2501 | CCOc(cc1)cc(O2)c1C=C(C(Nc1ccc(C)cc1)=O)C2=O | -8.021 |
| G265-0023 | CC(SC1=NC(COC(COc2c(C)cccc2)=O)=C2)=NN1C2=O | -7.939 |
| Y507-3071 | CCOC(c(cc1)ccc1N(C(C1)=O)N=C1C(F)(F)F)=O | -7.905 |
| Y501-8506 | CCOC(c1c(NC(c(cc2)cc(OC)c2OC(F)F)=O)sc(C)c1C)=O | -7.891 |
| 8010-1780 | Cc(cc1)cc(C)c1C(OC1=O)=C/C1=C/c1ncccc1 | -7.805 |
| G264-0002 | CC(SC1=NC(COC(CNC(c2ccccc2)=O)=O)=C2)=NN1C2=O | -7.688 |
| 8016-9786 | Cc(cc1)ccc1N/N=C1\N=C(c2ccccc2)OC1=O | -7.473 |
| G265-0376 | O=C(c(cc1)ccc1Cl)OCC(N=C1SC=CN11)=CC1=O | -7.425 |
| G265-0935 | COCCC(SC1=NC(COC(c2cccs2)=O)=C2)=NN1C2=O | -7.403 |
| 7541-0103 | CCOC(C(C=C1NC(c(cc2)ccc2OC)=O)=C2Oc(cccc3)c3N2C1=O)=O | -7.373 |
| G265-0400 | O=C(COc(cccc1)c1F)OCC(N=C1SC=CN11)=CC1=O | -6.959 |
| D443-0723 | CCOC(c(cccc1)c1NC(C1=Cc2cc(Cl)cc(OC)c2OC=C1)=O)=O | -6.922 |
| Y021-1998 | CCOC(C1=CNC(N(CC2)Cc3c2cccc3)=NC1=O)=O | -6.881 |
| 2124-0408 | COc(ccc(/C=C1/N=C(c2cccc([N+]([O-])=O)c2)OC1=O)c1)c1OC | -6.863 |
| 8013-2014 | COc(c(OC)c1)cc2c1N=C(/C=C/c1cccc([N+]([O-])=O)c1)OC2=O | -6.827 |
| Y200-6507 | CCOc1cccc(C=C2C(Nc3c(C)ccc(F)c3)=O)c1OC2=O | -6.700 |
| G265-0441 | CC(N12)=CSC2=NC(COC(c2ccc(C)cc2)=O)=CC1=O | -6.562 |
| 5639-0175 | COc(ccc(C(OC1=O)=C/C1=C/c(cccc1)c1[N+]([O-])=O)c1)c1OC | -6.472 |
| Y021-7068 | CCOC(C1=CC(C(CC(C2)c3ccccc3)=O)=C2NC1=O)=O | -6.288 |
| V021-5095 | CCOC(C(C1=O)=C(Nc2cc(F)ccc2)S/C1=C/c(cc1)cc2c1OCO2)=O | -6.148 |
| 2192-1317 | [O-][N+](c(cc1)cc(C(OC2=O)=N/C2=C\c(cc2)cc3c2OCO3)c1Cl)=O | -6.074 |
| 2125-0562 | COc(ccc(Cl)c1)c1C(OC1=O)=N/C1=C\c(cc1)cc2c1OCO2 | -6.038 |
| 1319-0181 | Cc1cccc(NC(C2=Cc3cccc(OC)c3OC2=O)=O)c1 | -5.989 |
| G786-0340 | CCOC(C(S1)=C(C)N(C)/C1=N/C(c(cc1)cc2c1OCCO2)=O)=O | -5.843 |
| 2113-0166 | COc(ccc(C(OC1=O)=N/C1=C\c(cc1)cc(OC)c1OC)c1)c1[N+]([O-])=O | -5.787 |
| G265-0143 | CCC(SC1=NC(COC(c2c(C)cccc2)=O)=C2)=NN1C2=O | -5.594 |
| G265-0191 | CC(C)CC(SC1=NC(COC(c2cccs2)=O)=C2)=NN1C2=O | -5.402 |
| G265-0325 | CC(C)(C)C(OCC(N=C1SC(C2CC2)=NN11)=CC1=O)=O | -5.101 |
| Y510-7248 | CCOC(c(cc1)ccc1N(C1=O)N=C(C(F)(F)F)/C1=C/c1cccs1)=O | -4.924 |
| G265-0503 | CC(SC1=NC(COC(c2ccc(C)cc2)=O)=C2)=CN1C2=O | -4.893 |
| K088-1750 | COc(ccc(C(OC1=O)=N/C1=C/c(cccc1)c1Cl)c1)c1OC | -3.908 |
| 1501-1398 | CCOC(C(C(c1cc([N+]([O-])=O)ccc1)N1)=C(C)NC1=S)=O | -3.703 |
| K088-1418 | COc(ccc([N+]([O-])=O)c1)c1C(OC1=O)=N/C1=C/c(cc1)cc(OC)c1OC | -3.501 |
| 3720-0235 | COc1cccc(C=C2C(Nc(cc3)ccc3[N+]([O-])=O)=O)c1OC2=O | -3.389 |
| G429-0006 | CC(OCc1cnc(C)c(O2)c1C=C(C(Nc1cc(C)ccc1)=O)C2=O)=O | -2.917 |
| 2124-0406 | CC(Oc(ccc(/C=C1/N=C(c2cccc([N+]([O-])=O)c2)OC1=O)c1)c1OC)=O | -2.217 |
| G265-0172 | CCC(SC1=NC(COC(c2cc(F)ccc2)=O)=C2)=NN1C2=O | 0.548 |


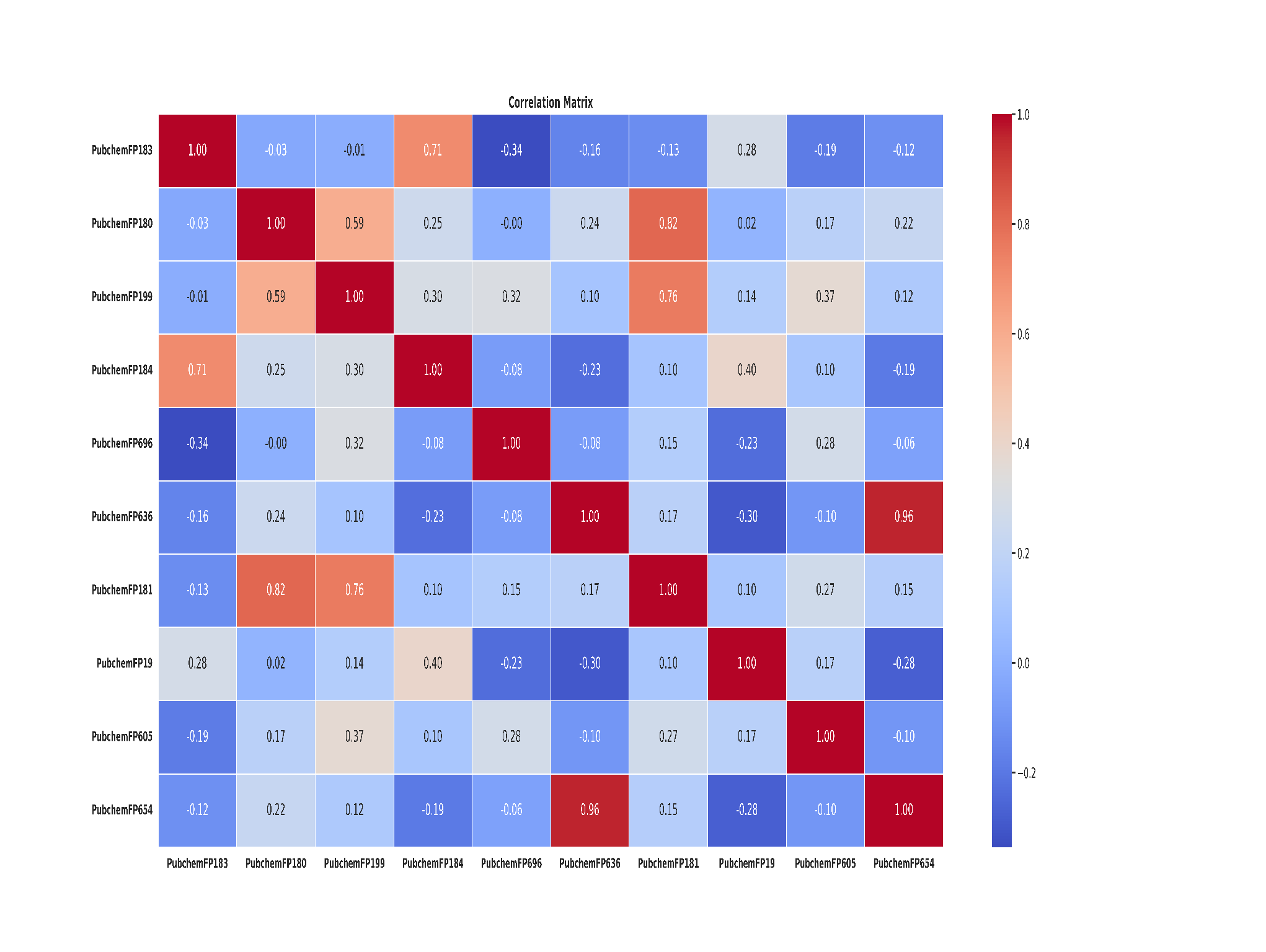


**Figure S1.** Correlation matrix for the best 10 structural features obtained through VIT plot analysis of PubChem fingerprint prediction model


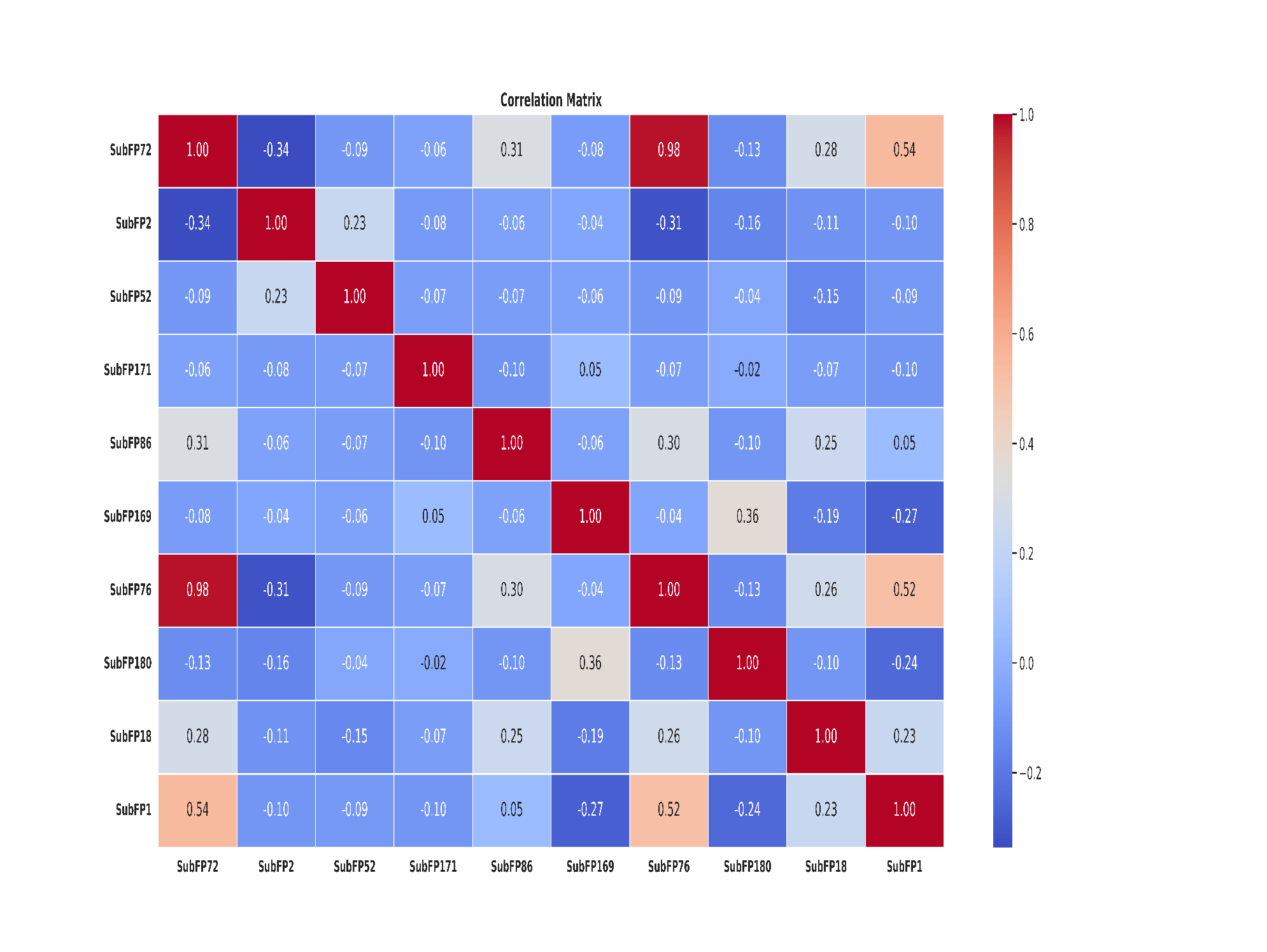


**Figure S2.** Correlation matrix for the best 10 structural features obtained through VIT plot analysis of substructure fingerprint prediction model


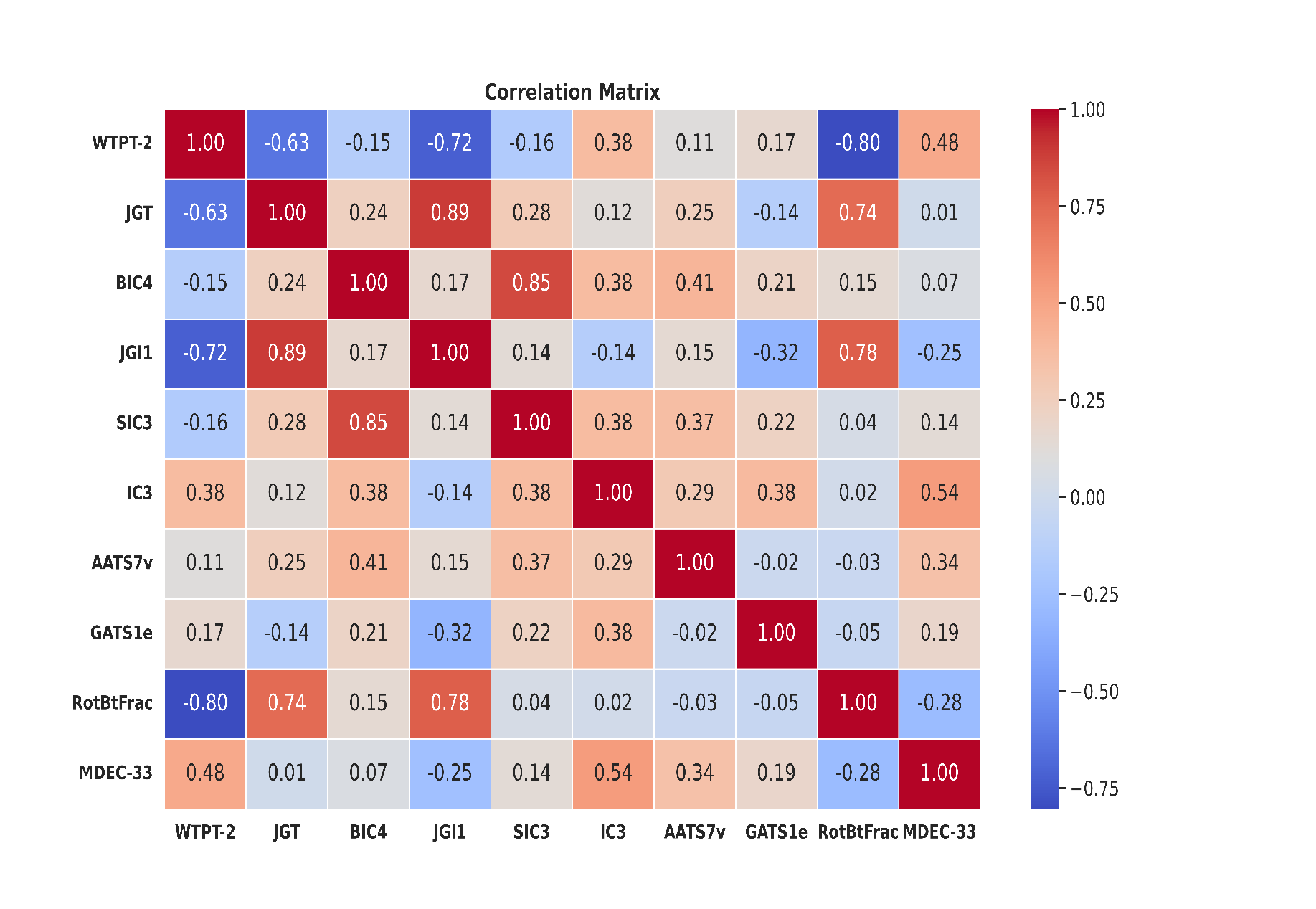


**Figure S3.** Correlation matrix for the best 10 structural features obtained through VIT plot analysis of 1D 2D molecular descriptor prediction model
